# Sarcomeric deficits underlie MYBPC1-associated myopathy with myogenic tremor

**DOI:** 10.1172/jci.insight.147612

**Published:** 2021-10-08

**Authors:** Janelle Geist Hauserman, Janis Stavusis, Humberto C. Joca, Joel C. Robinett, Laurin Hanft, Jack Vandermeulen, Runchen Zhao, Joseph P. Stains, Konstantinos Konstantopoulos, Kerry S. McDonald, Christopher Ward, Aikaterini Kontrogianni-Konstantopoulos

**Affiliations:** 1 Department of Biochemistry and Molecular Biology, University of Maryland School of Medicine, Baltimore, Maryland, USA.; 2 Latvian Biomedical Research and Study Centre, Riga, Latvia.; 3 Department of Orthopedics, University of Maryland School of Medicine, Baltimore, Maryland, USA.; 4 Department of Medical Pharmacology and Physiology, University of Missouri, School of Medicine Columbia, Missouri, USA.; 5 Department of Chemical and Biomolecular Engineering, Johns Hopkins University, Baltimore, Maryland, USA.

**Keywords:** Cell Biology, Muscle Biology, Cytoskeleton, Muscle, Neuromuscular disease

## Abstract

Myosin binding protein-C slow (sMyBP-C) comprises a subfamily of cytoskeletal proteins encoded by *MYBPC1* that is expressed in skeletal muscles where it contributes to myosin thick filament stabilization and actomyosin cross-bridge regulation. Recently, our group described the causal association of dominant missense pathogenic variants in *MYBPC1* with an early-onset myopathy characterized by generalized muscle weakness, hypotonia, dysmorphia, skeletal deformities, and myogenic tremor, occurring in the absence of neuropathy. To mechanistically interrogate the etiologies of this *MYBPC1*-associated myopathy in vivo, we generated a knock-in mouse model carrying the E248K** pathogenic variant. Using a battery of phenotypic, behavioral, and physiological measurements spanning neonatal to young adult life, we found that heterozygous E248K mice faithfully recapitulated the onset and progression of generalized myopathy, tremor occurrence, and skeletal deformities seen in human carriers. Moreover, using a combination of biochemical, ultrastructural, and contractile assessments at the level of the tissue, cell, and myofilaments, we show that the loss-of-function phenotype observed in mutant muscles is primarily driven by disordered and misaligned sarcomeres containing fragmented and out-of-register internal membranes that result in reduced force production and tremor initiation. Collectively, our findings provide mechanistic insights underscoring the E248K-disease pathogenesis and offer a relevant preclinical model for therapeutic discovery.

## Introduction

Myosin binding protein-C (MyBP-C) comprises a family of proteins expressed in striated muscles with structural and regulatory roles (1). Three distinct isoforms have been characterized, including the cardiac (c), fast (f) skeletal, and slow (s) skeletal proteins (1). sMyBP-C is encoded by *MYBPC1* located on chromosome 12 in humans (2), and it is heavily spliced, giving rise to a subfamily of at least 14 variants that are coexpressed in different amounts and combinations in both slow- and fast-twitch skeletal muscles (3).

The NH_2_-terminus of sMyBP-C supports binding to actin and myosin subfragment-2 (S2) within the C-zone of the A-band in a variant-specific manner (4). Dominant missense and recessive nonsense pathogenic variants in *MYBPC1* located in the NH_2_ and COOH termini were previously associated with the development of severe and lethal forms of distal arthrogryposis (DA) (5–10). Recently, dominant missense pathogenic variants residing within the NH_2_-terminal M-motif of sMyBP-C have been causatively linked to a new form of early-onset myopathy characterized by hypotonia, muscle weakness, dysmorphia, skeletal deformities, and a posturally pronounced, high-frequency tremor, likely of myogenic origin (11, 12).

In particular, the heterozygous E248K pathogenic variant was identified in a multigeneration family from Latvia and categorized as highly deleterious with a combined annotation dependent depletion (CADD) score of 36 (11). All carriers, 2 males and 2 females, exhibited hypotonia and hand tremor since infancy, as well as delayed gross motor milestone acquisition with independent walking achieved at 2 years of age (11). In early adolescence, the symptoms progressed to generalized muscle weakness, postural tremor, scoliosis, and thoracic asymmetry (11). While the rapid deterioration in muscle strength was stabilized in late adolescence, patients reported frequent respiratory issues, likely due to weakness of the respiratory musculature and altered thoracic structure (11). In adulthood, clinical findings include axial muscle weakness; proximal appendicular weakness; scoliosis with thoracic/sternal deformity; rigidity of the spine; contractures in the elbows, arms, and/or feet; and high-frequency tremor (~10 Hz) of irregular amplitude that is accentuated by posture and/or activity and is more noticeable in the hands than the legs (11).

Latvian patients heterozygous for the E248K pathogenic variant continually declined biopsies at clinic visits, which limited molecular insight into the pathology of the myopathy. Thus, initial mechanistic clues about the disease etiology came from biochemical and modeling studies from our group, and they indicated significantly enhanced binding between recombinant sMyBP-C carrying the E248K variant and myosin, most likely due to increased electrostatic interactions (11). While these results suggest structural and/or contractile deficits as the basis of the disease pathogenesis, in vivo evidence is lacking.

To gain insights into the mechanisms by which the E248K pathogenic variant drives disease pathogenesis, we generated a knock-in (KI) mouse model of this variant. We show that the heterozygous E248K KI model faithfully recapitulates the myopathic phenotype, tremor occurrence, and disease onset and progression seen in human E248K carriers. Moreover, in both slow- and fast-twitch skeletal muscles, we link the heterozygous presence of the E248K variant to a loss in muscle contractility underscored by disordered sarcomere structure and compromised function.

## Results

**Figure 1:**
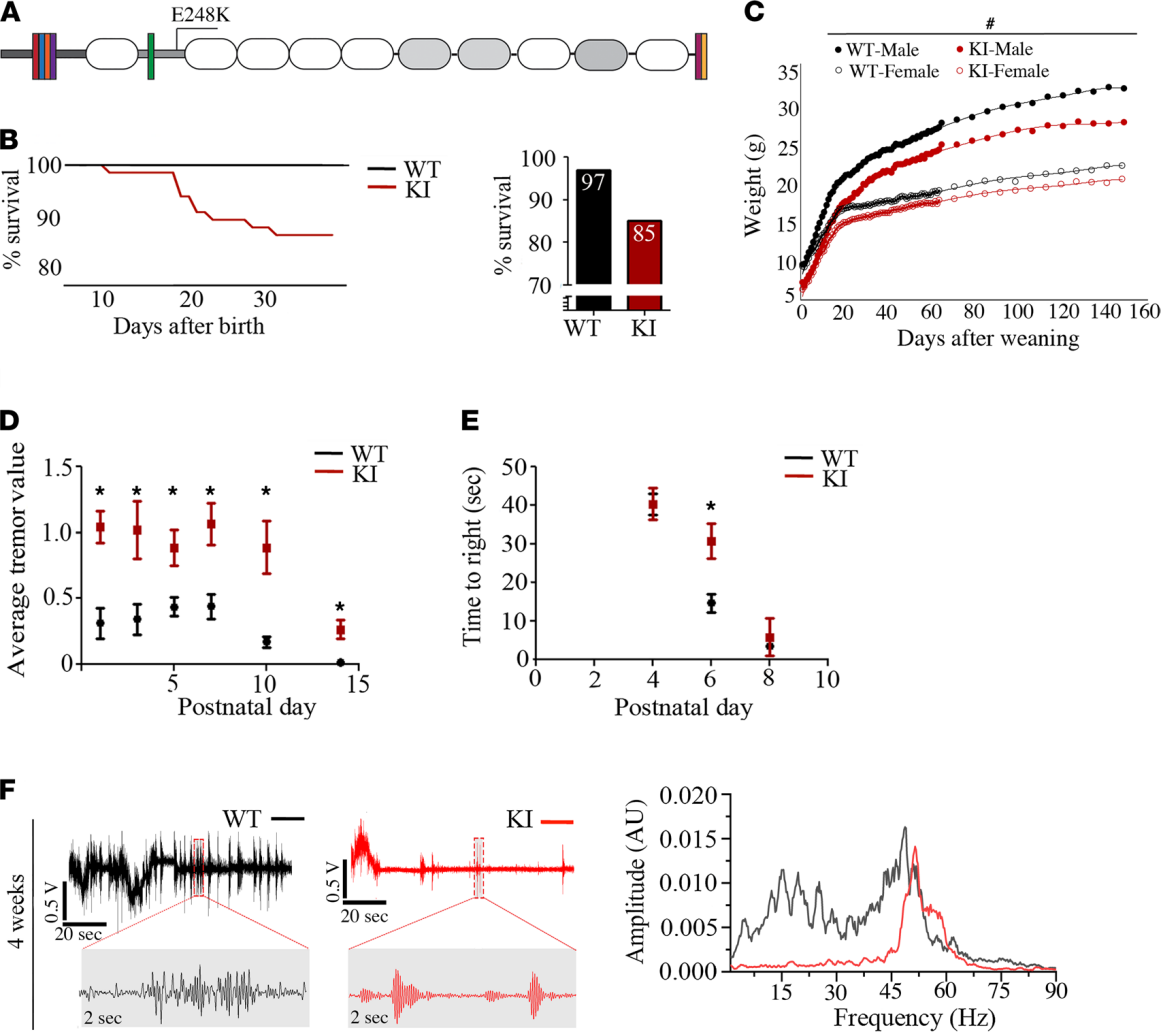
Generation and physiological characterization of the *MYBPC1* E248K KI mouse model. (**A**) Schematic representation of the sMyBP-C domain structure and location of the E248K variant in the M-motif; white and gray ovals denote immunoglobulin and fibronectin-III domains, dark gray and light gray horizontal rectangles indicate the Pro/Ala and M-motif, and colored rectangles depict spliced sequences. (**B**) Approximately 15% of heterozygous KI mice failed to survive through weaning but showed similar survival trends as WT past 3 weeks of age; survival chart: *n* = 295 WT (151 male and 144 female) and *n* = 154 heterozygous KI (80 male and 74 female). The overall survival bar-graph shows: *n* = 97 WT (52 male and 45 female) and *n* = 65 heterozygous KI (37 male and 28 female). (**C**) Both male and female heterozygous KI mice were significantly smaller compared with WT littermates through 24 weeks of age; *n* = 22 WT (10 male and 12 female) and *n* = 24 heterozygous KI (13 male and 11 female). (**D**) During the first 15 days of postnatal life, average tremor value at rest was significantly higher in heterozygous KI pups compared with WT; *n* = 14 WT (4 male and 10 female) and *n* = 9 heterozygous KI (6 male and 3 female). (**E**) Heterozygous KI pups needed significantly longer time to right than WT at P6; *n* = 27 WT and *n* = 16 heterozygous KI. (**F**) High-frequency tremor was identified in 92% (12 of 13) of heterozygous KI mice at 4 weeks compared with 10% (1 of 10) of WT using a custom-designed vibration plate. Representative tremor frequency traces are shown, indicating random movement in WT and bursts of tremor in heterozygous KI animals; areas marked with red dashed boxes are blown up for comparison and detailed evaluation; *n* = 10 WT (6 male and 4 female) and *n* = 13 heterozygous KI (6 male and 7 female). Statistical significance was calculated with a 2-tailed Student’s *t* test for all applicable assays. ^#^*P* < 0.05, *P < 0.01.

Using CRISPR/Cas9 technology, we generated a KI mouse model carrying the *MYBPC1* E248K pathogenic variant (Figure 1A), which is causatively linked to generalized myopathy with myogenic tremors in both male and female members of a Latvian family (11). KI mice homozygous for the E248K variant exhibit severe head and body tremor (Supplemental Video 1; supplemental material available online with this article; https://doi.org/10.1172/jci.insight.147612DS1), display signs of dyspnea and cyanosis, and die shortly after birth. In contrast are heterozygous KI mice that typically survive to adulthood, with only a small percentage (~15%) dying before weaning at 3 weeks (Figure 1B); of note, examination of the relative expression of the WT and E248K alleles in 4-week heterozygous KI extensor digitorum longus (EDL) and soleus muscles using droplet digital PCR (ddPCR) indicated a normalized fractional abundance value of 1 for WT and ~0.5 for KI muscles, which is consistent with the heterozygous expression of the variant allele in our KI model (Supplemental Figure 1A). Compared with WT, heterozygous KI mice are visibly smaller throughout postnatal development and exhibit decreased body mass through maturity (Figure 1C), despite normal feeding ability. Similar to humans heterozygous for the E248K variant (11), heterozygous KI mice display intense head and body tremor during postnatal development both at rest (Figure 1D) and during locomotion (Supplemental Videos 2–12), in addition to a significant latency in righting reflex at P6 (Figure 1E). As in human carriers (11), tremor occurrence in heterozygous KI mice is diminished in adulthood, arising with intention or posture acquisition, as evidenced in a hindlimb-clasping assay (Supplemental Videos 13 and 14).

In affected humans, CNS adaptation of motor unit control is thought to decrease tremor occurrence in adulthood (13, 14). In this regard, we used light sedation to suppress CNS-dependent motor control in 4-week-old mice. During anesthesia recovery, we monitored for tremor occurrence on a custom platform supported by a force displacement transducer (GRASS FT-03). We found that 92% (12 of 13) of heterozygous KI versus 10% (1 of 10) of WT mice exhibited distinct tremors defined by periodic bursts of muscle activity eliciting platform vibration at 55–65 Hz (Figure 1F). This frequency response aligns with the allometric relationship between body mass and tremor/shiver frequency, with humans and larger animals displaying low frequency (8–15 Hz) and smaller animals displaying higher-frequency (30–60 Hz) events (15).

**Figure 2:**
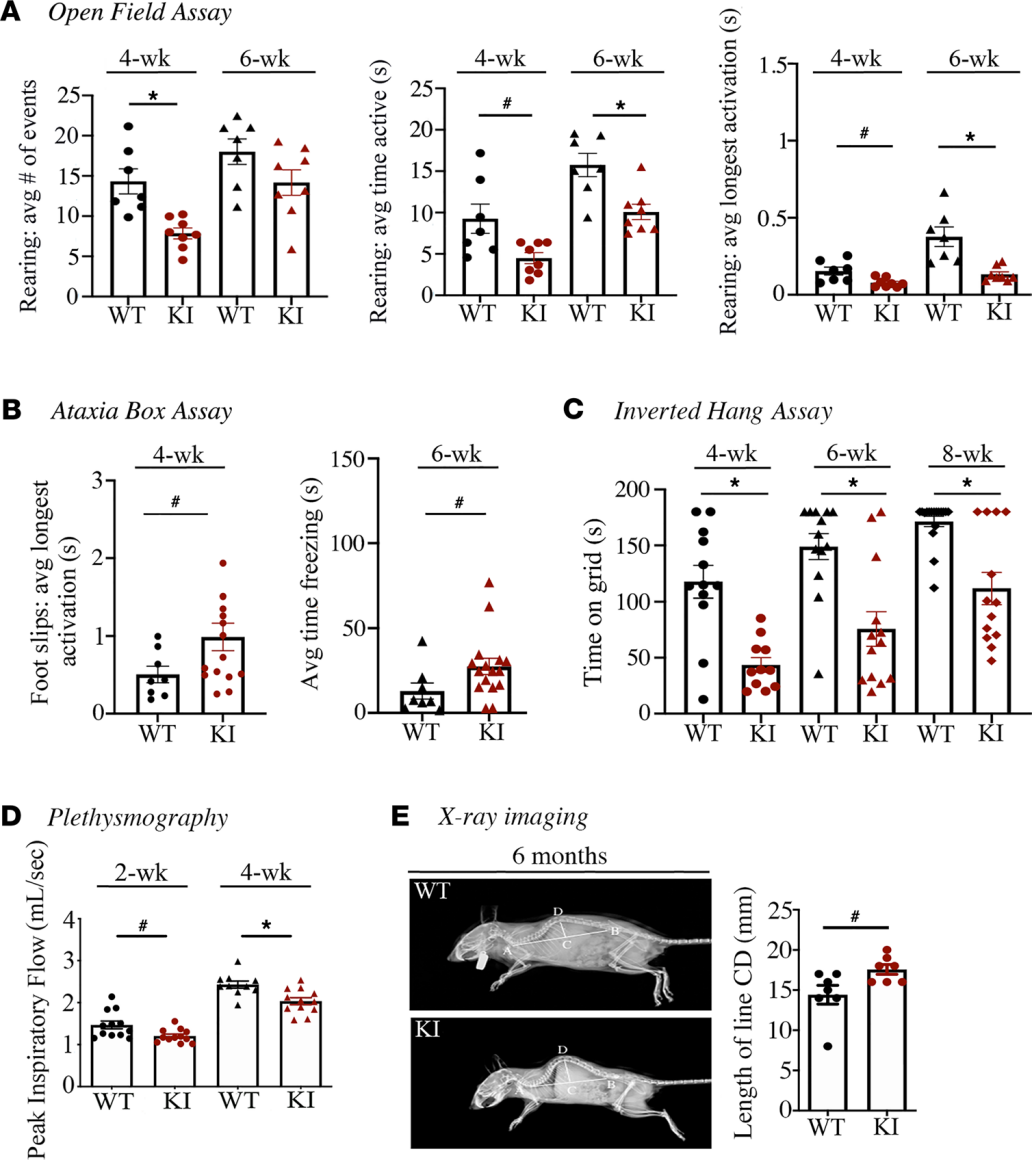
Behavioral and phenotypic assessment of heterozygous E248K KI mice. (**A**) Young adult heterozygous KI mice performed significantly fewer rearing events and spent less time rearing in an open field assay compared with WT littermates; all ages, *n* = 7 WT (2 male and 5 female) and *n* = 8 heterozygous KI (2 male and 6 female). (**B**) Young adult heterozygous KI mice exhibited significantly more foot slips and increased time freezing or time immobile in an ataxia box assay compared with WT littermates; all ages, *n* = 8 WT (5 male and 3 female) and *n* = 15 heterozygous KI (8 male and 7 female). (**C**) Young adult heterozygous KI mice displayed significantly decreased overall grip and body strength during an inverted hang assay compared with WT littermates, as evidenced by significantly less time on the grid; 4 weeks, *n* = 12 WT (7 male and 5 female) and *n* = 11 heterozygous KI (6 male and 5 female); 6 weeks, *n* = 13 WT (8 male and 5 female) and *n* = 13 heterozygous KI (7 male and 6 female); and 8 weeks, *n* = 17 WT (9 male and 8 female) and *n* = 13 heterozygous KI (7 male and 6 female). (**D**) Plethysmography analysis revealed significant deficits in peak inspiratory flow for heterozygous KI animals at 2 and 4 weeks of age; 2 weeks, *n* = 12 WT (6 male and 6 female) and *n* = 12 heterozygous KI (5 male and 7 female), and 4 weeks, *n* = 10 WT (5 male and 5 female) and *n* = 11 heterozygous KI (6 male and 5 female). (**E**) Representative radiographs of 6-month-old WT and heterozygous KI mice and kyphotic index quantification revealed the presence of significant kyphosis in heterozygous KI animals; *n* = 7 WT (4 male and 3 female) and *n* = 7 heterozygous KI (4 male and 3 female). Statistical significance was calculated with a 2-tailed Student’s *t* test for animals at the same age. ^#^*P* < 0.05, *P < 0.01.

To determine if heterozygous KI mice develop generalized myopathy, we evaluated age-matched KI and WT littermates against a battery of behavioral and functional assessments in vivo. In an open field assay quantifying voluntary activity, heterozygous KI mice exhibited a significant reduction in the number and time of rearing events at 4 and 6 weeks of age (Figure 2A). Aligned with their reduced locomotor activity, an ataxia box assay evaluating balance, motor coordination, and movement revealed that heterozygous KI mice displayed significantly more foot slips at 4 weeks and increased episodes of immobility/freezing at 6 weeks (Figure 2B). Using a 4-limb inverted hang test, we further found that 4-, 6-, and 8-week-old heterozygous KI mice exhibited significantly reduced hang-time on the grid compared with age-matched WT, indicative of decreased grip and whole body strength/endurance (Figure 2C and Supplemental Videos 15 and 16). Taken together, the decreased mobility and generalized muscle weakness identified in the heterozygous E248K mice in vivo aligns with the reduced neuromuscular function in patients heterozygous for the E248K pathogenic variant (11).

An increased incidence of respiratory illness is pathognomonic in patients heterozygous for the E248K variant, with links to thoracic deformity and respiratory muscle weakness (11). Assessment of respiratory muscle function using whole body plethysmography showed that heterozygous KI mice have significant deficits in peak inspiratory flow (PIF) at 2 and 4 weeks of age compared with WT (Figure 2D). Moreover, x-ray imaging revealed significant kyphosis in 6-month-old heterozygous KI mice (Figure 2E), consistent with thoracic and sternal deformities, scoliosis, and rigidity of the spine observed in E248K human carriers (11).

**Figure 3:**
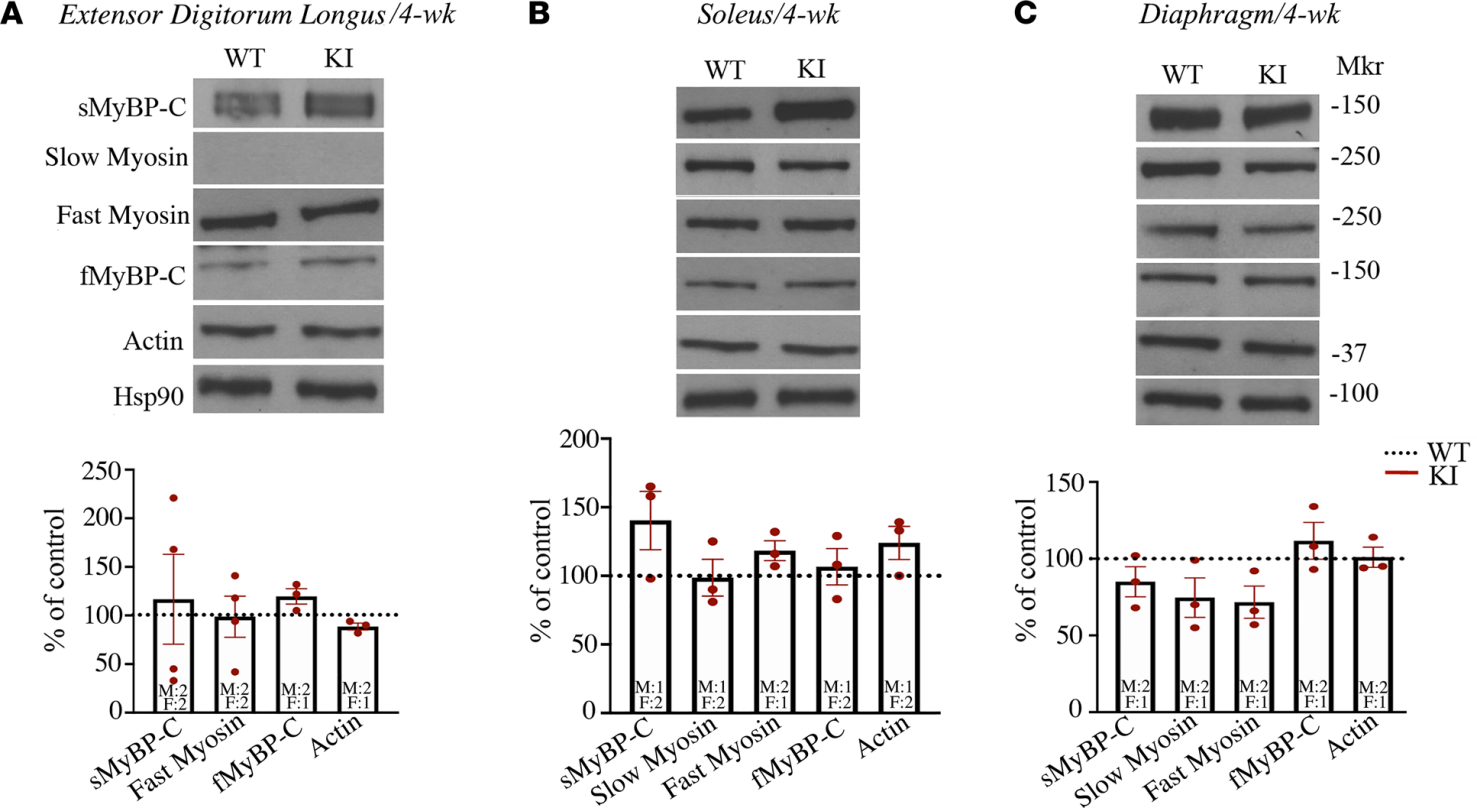
Biochemical analysis of heterozygous KI muscles at 4 weeks. (**A**–**C**) Representative immunoblots and quantification of the percent expression of major myofibrillar proteins in heterozygous KI EDL (**A**), soleus (**B**), and diaphragm (**C**) muscles indicated statistically unaltered protein expression at 4 weeks of age. All values are compared with WT, which was set to 100% following normalization to Hsp90 that was used as a loading control. All blots were processed in parallel. Individual data points represent the number of biological samples evaluated for each protein obtained from both male (M) and female (F) mice, as indicated in the dot plots. Statistical significance was calculated with a normalized 2-tailed Student’s *t* test.

We sought mechanistic insights into these in vivo functional deficits with biochemical, morphometric, and ex vivo contractility assays. Importantly, we profiled both slow- and fast-twitch muscles, as the E248K variant is present in all known sMyBP-C transcripts, expressed in variable amounts and combinations across slow- and fast-twitch fibers. Immunoblot analysis demonstrated similar levels of total sMyBP-C in WT and heterozygous KI fast-twitch EDL, slow-twitch soleus, and mixed-fiber diaphragm muscles at both 2 (Supplemental Figure 1, B–D) and 4 (Figure 3, A–C) weeks of age. Further examination of the expression profile of additional sarcomeric proteins including slow and fast myosin, fMyBP-C, and actin did not reveal any significant alterations in heterozygous KI EDL, soleus, and diaphragm muscles compared with WT either at 2 (Supplemental Figure 1, B–D) or 4 (Figure 3, A–C) weeks of age; of note, slow myosin was undetectable in both WT and heterozygous KI EDL muscles at either 2 or 4 weeks.

**Figure 4:**
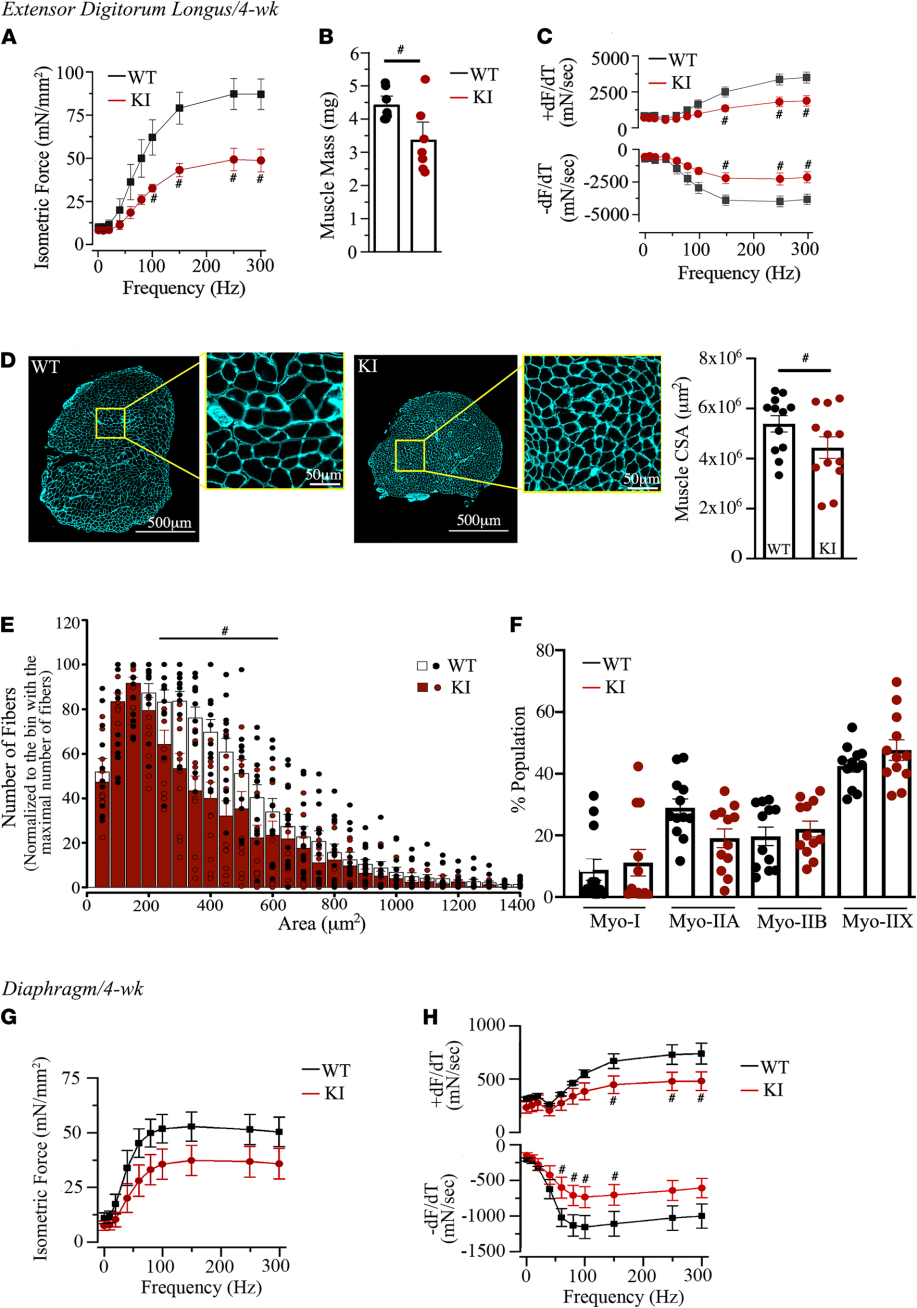
Functional and histological evaluation of heterozygous KI EDL muscles at 4 weeks. (**A**–**C**) Heterozygous KI EDL muscle exhibited significantly reduced peak isometric force production (**A**) that persisted after normalization for the decrease in mass (**B**), and reduced contraction and relaxation velocities at higher frequencies (**C**); *n* = 8 WT (7 male and 1 female) and *n* = 7 heterozygous KI (5 male and 2 female). Statistical significance was calculated with repeated measures 1-way ANOVA (**A** and **C**) or Mann-Whitney *U* (**B**) tests. (**D**) Representative cross-sectional images of WT and heterozygous KI EDL muscles stained for laminin (membrane marker; teal pseudocolor). Whole muscle CSA of heterozygous KI EDL muscles was significantly decreased compared with WT; *n* = 4 WT (2 male and 2 female) and *n* = 4 heterozygous KI (2 male and 2 female). Data points represent 3 cross-sectional measurements per muscle along the longitudinal axis of the tissue to account for innate variation between the middle and ends. Statistical significance was calculated with 1-tailed Student’s *t* test. Scale bars: 500 µm, inset 50 µm. (**E**) Plotting of fiber size distribution indicated that heterozygous KI EDL muscles contained significantly fewer myofibers with CSA of 300–600 μm^2^ compared with WT; *n* = 3 WT (2 male and 1 female) and *n* = 3 heterozygous KI (2 male and 1 female). Statistical significance was calculated with a Mann-Whitney *U* test. (**F**) Quantification of fiber type population did not indicate significant differences between heterozygous KI and WT EDL muscles; *n* = 3 WT (2 male and 1 female) and *n* = 3 heterozygous KI (2 male and 1 female). Statistical significance was calculated with Kruskal-Wallis test. (**G** and **H**) Heterozygous KI diaphragm strips exhibited a trend toward decreased force (**G**), and significantly reduced contraction and relaxation velocities at high and mid-range frequencies (**H**), respectively; *n* = 8 WT (7 male and 1 female) and *n* = 7 heterozygous KI (5 male and 2 female). Statistical significance was calculated with repeated measures 1-way ANOVA. ^#^*P* < 0.05, *P < 0.01.

We next quantified the in vitro function of EDL and diaphragm muscles explanted from 4-week-old mice by examining the force versus frequency relationship (500 msec trains of 0.1 msec pulses at 1–300 Hz) using our established methods (16). We identified a significant deficit in isometric force in the KI EDL muscle (Figure 4A) despite normalization to the calculated muscle cross-sectional area (CSA), which accounts for the reduced EDL mass in the KI mice (Figure 4B). We further identified significant deficits in the rates of contraction (+dF/dT) and relaxation (–dF/dT) (Figure 4C) in the heterozygous KI EDL muscle. Taken together, these findings suggest intrinsic deficits in KI EDL muscle fiber function, independent of the deficits in muscle mass. Histological examination revealed that the decrease in mass in the heterozygous KI EDL muscle was accompanied by reduced whole muscle CSA (Figure 4D) driven by a decrease in the CSA of individual myofibers (Figure 4E), measured as in refs. 17 and 18. Notably, these alterations occurred independently of any change in muscle fiber type (Figure 4F). Similar to KI EDL, KI diaphragm also displayed significant deficits in mass (Supplemental Table 1). Moreover, dissected diaphragm strips from 4-week-old heterozygous KI mice showed a trend toward decreased normalized isometric force production (Figure 4G) and significantly reduced rates of contraction (+dF/dT) and relaxation (–dF/dT) at high- and midrange frequencies, respectively (Figure 4H). Of note, in addition to decreased EDL and diaphragm muscle weights, profiling of several other muscle groups revealed similar deficits in mass (Supplemental Table 1). Together, these results suggest that both decreased muscle mass and intrinsic changes in muscle fiber contractile function underly the deficits in neuromuscular performance in the heterozygous KI E248K mouse.

Given that we observed deficits in muscle function independent of its mass, we sought mechanisms within the muscle fiber to account for the observed loss-of-function phenotype. Our initial focus was on evidence indicating that cMyBP-C sensitizes the contractile apparatus to Ca^2+^ activation by counterbalancing the spatiotemporal gradient of Ca^2+^ release to yield synchronous cross-bridge activation (19). Our assessment of electrically evoked Ca^2+^ kinetics in WT and heterozygous KI EDL muscles found a significant increase in the magnitude of Ca^2+^ release (Supplemental Figure 2A), accompanied by a decreased rate of Ca^2+^ release (Supplemental Figure 2B) and prolonged Ca^2+^ uptake (Supplemental Figure 2C). While significant alterations were identified, they could not account for the deficits in contractility that were observed.

**Figure 5:**
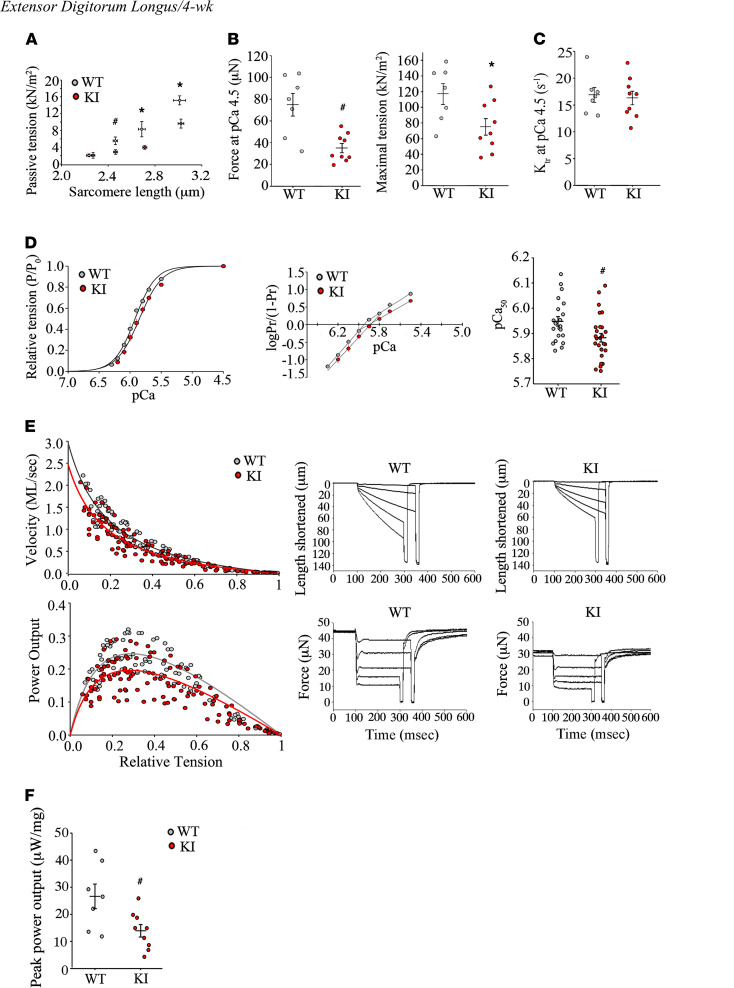
Functional alterations in heterozygous KI EDL myofibers at 4 weeks of age. (**A**–**F**) Single, permeabilized KI EDL myofibers exhibited decreased passive tension (**A**), maximal Ca^2+^-activated force and tension (**B**), Ca^2+^ sensitivity of force (**D**), loaded shortening velocity (**E**), and power output (**F**), independent of any change in the rate of tension redevelopment (max *k_tr_*) (**C**). For **D**, 3 pCa_50_ values were calculated for each myofiber, one from the tension-pCa curve and the other 2 from the 2 *x*-intercepts of the linearized tension-pCa fits (44); *n* = 7 WT fibers obtained from 5 male and 2 female animals and *n* = 9 heterozygous KI fibers obtained from 4 male and 5 female animals. Statistical significance was determined via a 2-tailed Student’s *t* test (**A**, **C**, **D**, and **F**) or 2-way ANOVA (**B**). ^#^*P* < 0.05, *P < 0.01.

We next focused on the myofilaments where extensive work has implicated MyBP-C proteins in the modulation of the cross-bridge cycle (20–23). Here, we sought to determine the impact of the E248K variant on Ca^2+^-dependent force generation in single, skinned EDL myofibers from 4-week-old WT and heterozygous KI mice using methods previously described (24, 25). In the relaxed state (i.e., no activating Ca^2+^), KI fibers exhibited significantly decreased passive tension quantified by the sarcomere length versus passive force relationship (Figure 5A). Following Ca^2+^ activation of the cross-bridge cycle, heterozygous KI fibers displayed significant deficits in maximal Ca^2+^-activated tension (Figure 5B), independent of any change in the rate of tension redevelopment (i.e., *k_tr_* values; Figure 5C), myofilament Ca^2+^ sensitivity (Figure 5D), loaded shortening velocity and normalized power output (Figure 5E), and peak absolute power output (Figure 5F) compared with WT. Given the decrease in maximal Ca^2+^ force independent of alterations in cross-bridge cycling kinetics, we considered sarcomere structural changes as a plausible explanation for the identified deficits in mutant myofibers.

**Figure 6:**
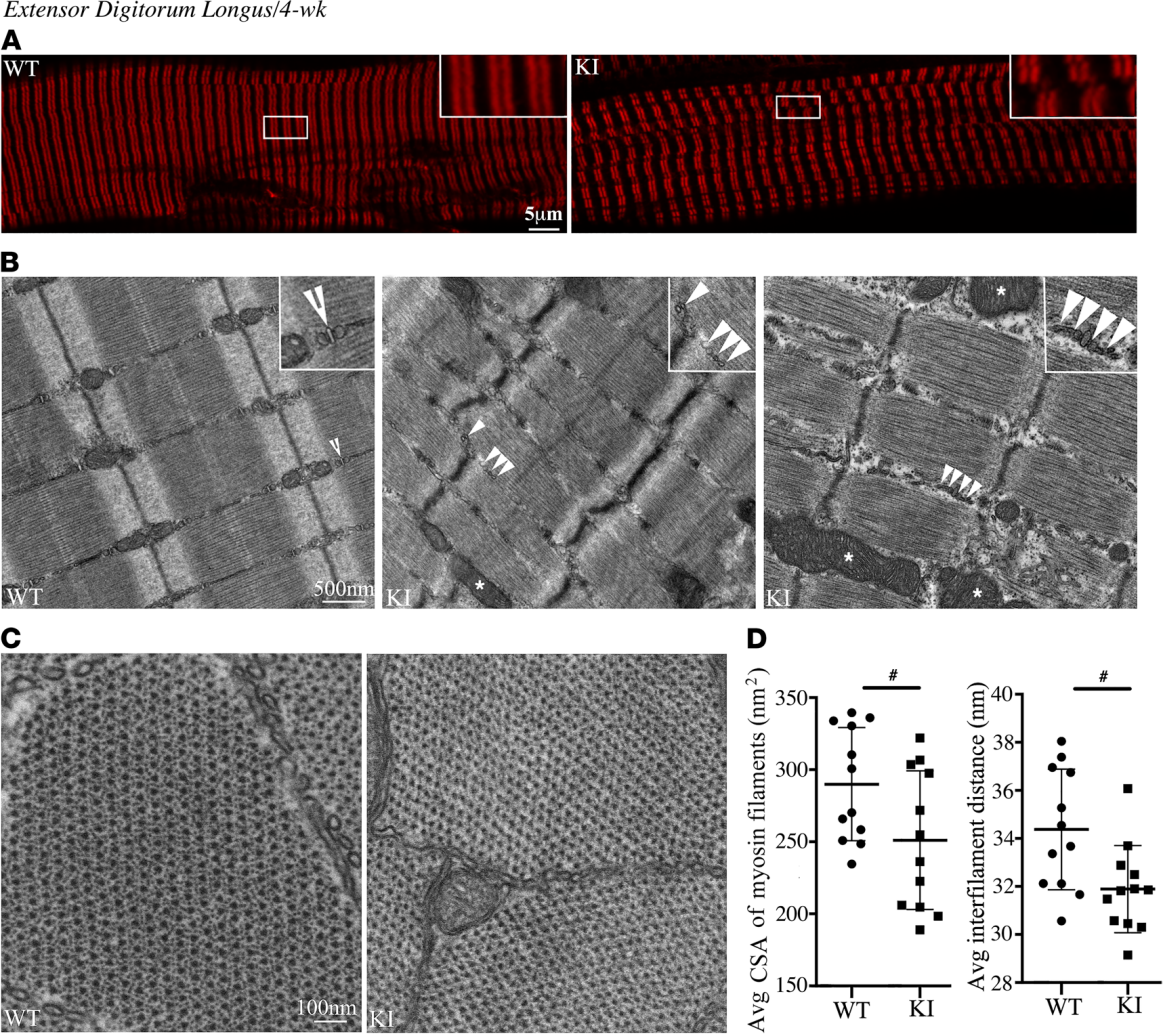
Structural evaluation of heterozygous KI EDL muscles at 4 weeks of age. (**A**) sMyBP-C assumed its typical distribution at the C-zone of A-bands in both WT and heterozygous KI EDL muscles; however, bundles of split myofibrils and misaligned sarcomeres were observed in heterozygous KI muscles; *n* = 4 WT (2 male and 2 female) and *n* = 4 heterozygous KI (2 male and 2 female). Scale bar: 5 µm. (**B**) Electron micrographs of longitudinal sections of WT EDL muscles confirmed the presence of properly organized and aligned sarcomeres with intact internal membranes forming typical triads (open arrowheads) at the level of A/I junctions. In contrast, heterozygous KI muscles exhibited less compact Z-discs and frequent Z-disc streaming; less defined A-, I-, and M-bands; enlarged and more abundant mitochondria (asterisks); and fragmented and/or misaligned internal membranes (closed arrowheads); *n* = 4 WT (2 male and 2 female) and *n* = 4 heterozygous KI (2 male and 2 female). Scale bar: 500 nm. (**C** and **D**) Cross-sectional electron micrographs of WT and heterozygous KI EDL muscles displayed normal hexagonal arrays of myosin filaments (**C**); however, the CSA of individual thick filaments and the interfilament distance was significantly decreased in the latter, as indicated by FFT analysis (**D**); *n* = 2 WT (1 male and 1 female) and *n* = 2 heterozygous KI (2 female). Scale bar: 100 nm. Two electron micrographs were analyzed per muscle by quantifying 3 randomly selected regions per micrograph including > 100 myofilaments. Statistical evaluation was performed with a 2-tailed unpaired *t* test (**D**). ^#^*P* < 0.05.

**Figure 7:**
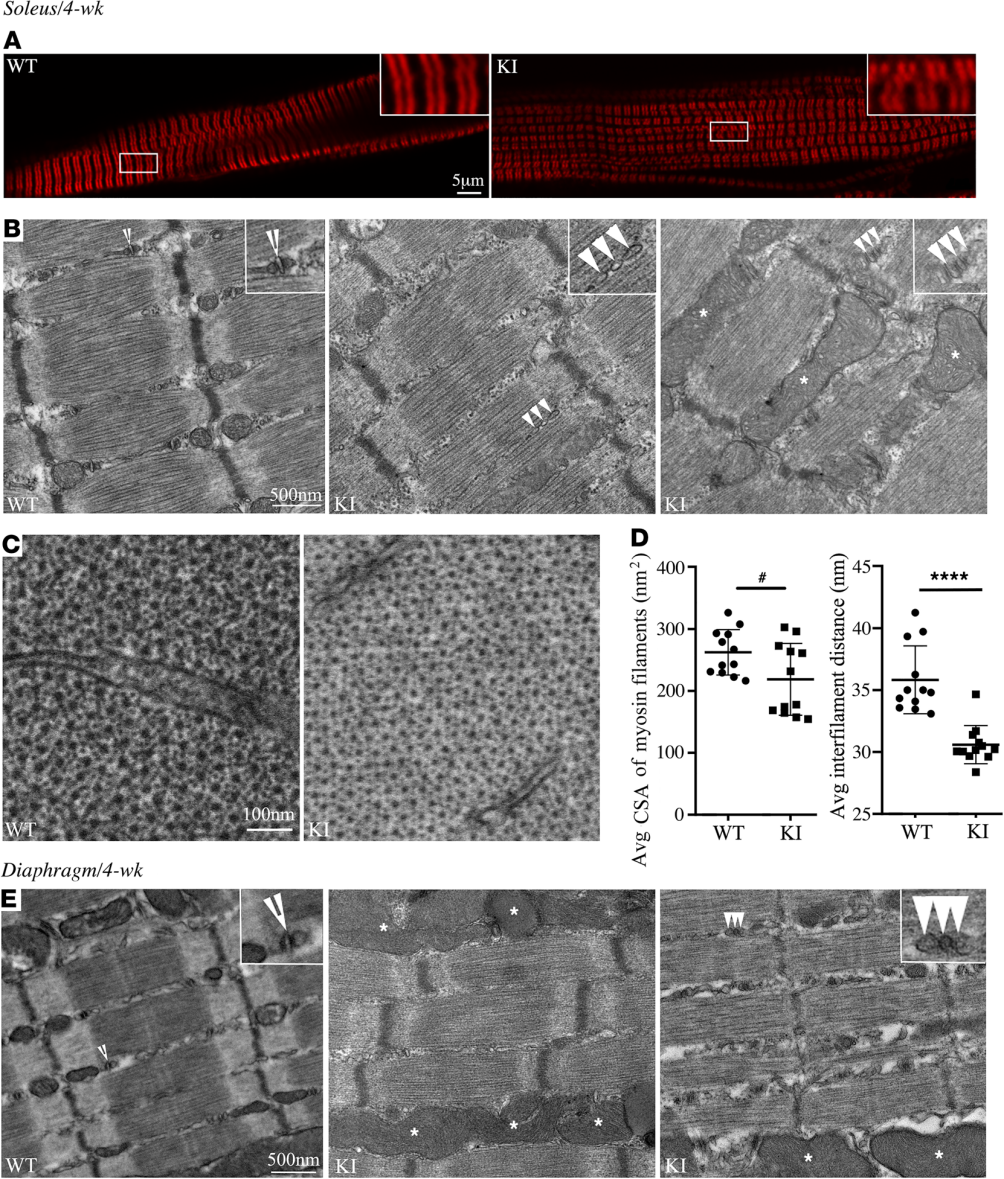
Structural evaluation of heterozygous KI soleus and diaphragm muscles at 4 weeks of age. (**A**) sMyBP-C exhibited its expected localization at the C-zone of the A-band in WT and heterozygous KI soleus muscles; however, similar to 4-week heterozygous KI EDL muscle, bundles of split myofibrils and misaligned sarcomeres were observed in the latter; *n* = 4 WT (2 male and 2 female) and *n* = 4 heterozygous KI (2 male and 2 female). Scale bar: 5 µm. (**B**) Electron micrographs of longitudinal sections of WT soleus muscles revealed the presence of structurally intact and registered sarcomeres with orderly internal membranes forming typical triads (open arrowheads) at the level of A/I junctions. In contrast, heterozygous KI soleus and diaphragm muscles displayed less compact Z-discs and frequent Z-disc streaming; hazy A-, I-, and M-bands; enlarged and more abundant mitochondria (asterisks); and fragmented and/or misaligned internal membranes (closed arrowheads); *n* = 4 WT (2 male and 2 female) and *n* = 4 heterozygous KI (2 male and 2 female). Scale bar: 500 nm. (**C** and **D**) Cross-sectional electron micrographs of WT and heterozygous KI soleus muscles contained typical hexagonal arrays of myosin filaments (**C**); however, both the thick filament CSA and interfilament distance were significantly reduced as quantified by FFT analysis (**D**); *n* = 2 WT (2 male) and *n* = 2 heterozygous KI (2 male). Scale bar: 100 nm. Two electron micrographs were analyzed per muscle by quantifying 3 randomly selected regions per micrograph including > 100 myofilaments. (**E**) Electron micrographs of longitudinal sections of WT and heterozygous KI diaphragm muscles showed similar structural alterations as those seen in heterozygous KI EDL and soleus muscles; *n* = 4 WT (2 male and 2 female) and *n* = 4 heterozygous KI (2 male and 2 female). Scale bar: 500 nm. Statistical significance was calculated with unpaired *t* test (D; area) or Mann Whitney *U* test (D; distance). ^#^*P* < 0.05, *P < 0.01, ****P < 0.0001.

Recent evidence has shown that sMyBP-C (1, 16) is critical to maintain sarcomere structure and, thus, myofiber integrity, which is unique compared with the cardiac and fast skeletal isoforms (20–23). To determine if the E248K pathogenic variant alters these functions, we first performed H&E staining of fast- (EDL) and slow-twitch (soleus) muscles at 2 and 4 weeks of age, and this did not reveal any gross morphological alterations between WT and KI muscles (Supplemental Figure 3). We then proceeded to examine the subcellular distribution of sMyBP-C in EDL and soleus muscles at 2 (Supplemental Figure 4, A and B) and 4 (Figure 6A and Figure 7A) weeks of age. We observed a regular organization of the protein in the C-zones of A-bands in WT and heterozygous KI muscles; however, bundles of split myofibrils and misaligned sarcomeres were observed in mutant muscles both at 2 and 4 weeks, consistent with a myopathic phenotype (26–28). Evaluation of the ultrastructural organization of WT and heterozygous KI EDL, soleus and diaphragm muscles substantiated these observations both during postnatal development at 2 weeks (Supplemental Figure 5, A, B, and G) and in early adulthood at 4 weeks (Figure 6B and Figure 7, B and E). Although the overall striated organization of the heterozygous KI muscles was preserved, close inspection of the ultrastructural organization of sarcomeres in longitudinal sections revealed less compact Z-discs, lack of clear distinction between A- and I-bands, and diffuse M-bands in all 3 muscles at both 2 and 4 weeks. Frequent Z-disk streaming was also observed in all 4-week heterozygous KI muscles compared with WT, as determined by the calculated percent occurrence of the phenotype (i.e., WT EDL, 25%; KI EDL, 100%; WT soleus, 0%; KI soleus, 100%; WT diaphragm, 75%; KI diaphragm, 100%); of note, Z-disk streaming was commonly observed in both WT and KI muscles at 2 weeks (i.e., WT EDL, 75%; KI EDL, 100%; WT soleus, 100%; KI soleus, 100%; WT diaphragm, 67%; KI diaphragm, 100%). In addition, enlarged and more abundant mitochondria (denoted with asterisks) were evident at 4 weeks (Figure 6B and Figure 7, B and E), which was corroborated by a significant increase in the average area occupied by mitochondria in KI EDL and soleus muscles compared with WT (Supplemental Figure 6A); similar evaluation at 2 weeks showed a trend toward increased mitochondrial area in both muscles (Supplemental Figure 6B), indicating progressive mitochondrial alterations between late postnatal development (2 weeks) and early adulthood (4 weeks). Moreover, contrary to WT muscles that contained typical triads positioned at A/I junctions (denoted with open arrowheads), KI muscles contained fragmented triads and/or misaligned internal membranes (denoted with closed arrowheads); this was more pronounced at 4 weeks (Figure 6B and Figure 7, B and E) compared with 2 weeks (Supplemental Figure 5, A, B, and G), indicating deterioration of internal membranes possibly as a function of aging. Further evaluation of cross sections of EDL and soleus muscles followed by Fast Fourier Transform (FFT) analysis revealed that the CSA of individual thick myosin filaments and their interfilament distance were significantly decreased in heterozygous KI EDL (Figure 6, C and D) and soleus (Figure 7, C and D) muscles compared with WT at 4 weeks. Interestingly, parallel evaluation of cross sections of EDL and soleus muscles at 2 weeks indicated markedly reduced CSA and interfilament distance of thick myosin filaments in heterozygous KI EDL (Supplemental Figure 5, C and E) but not soleus (Supplemental Figure 5, D and F) muscle, suggesting that mutant fast and slow twitch muscles may differentially progress in developing a myopathic phenotype, which is likely indicative of the increased susceptibility of fast-twitch muscles to stress.

## Discussion

We show that mice heterozygous for the E248K pathogenic variant in sMyBP-C faithfully model the disease phenotype reported in heterozygous E248K patients. These phenotypic traits include intense myogenic tremor at birth that diminishes in adolescence and skeletal muscle weakness that may impair neuromuscular function and predispose to skeletal/thoracic deformity to further impact respiratory function. Considering the lethality in homozygous KI mice, we suspect impaired respiratory function to be causal factors. As no homozygous E248K patients have been reported, it remains unknown if homozygosity contributes to neonatal or birth lethality in humans.

Placing these current findings in context with our evidence demonstrating (a) increased (>3-fold) binding of mutant sMyBP-C to myosin (11) and (b) the unique role of sMyBP-C in thick filament assembly and stabilization (16), we posit that this *MYBPC1* myopathy originates from alterations in the structure and, consequently, the regulation of myofilaments. To this end, our ultrastructural studies find shorter interfilament distances and more densely packed myofilaments in KI muscles, consistent with mutant sMyBP-C shifting closer to myosin heads. In this scenario, mutant sMyBP-C would potentially negatively impact sarcomeric organization and/or hinder effective actomyosin binding, underscoring the observed structural and contractile deficits. Consistent with this notion, we find malformed and misaligned myofibrils in KI myofibers from young mice (2 and 4 weeks of age), indicating that the structural role of sMyBP-C is impacted by the E248K pathogenic variant. Together, these findings are consistent with our earlier work demonstrating that downregulation of sMyBP-C in adult skeletal muscles results in distorted A-bands and reduced sarcomere length, resulting in decreased contractile kinetics at the level of intact myofibers and reduced twitch and tetanic force production at the level of whole muscles (16). This key structural role of sMyBP-C agrees with its early expression during embryonic development (29) and likely distinguishes it from its cardiac and fast skeletal counterparts that appear to primarily have regulatory roles (1, 19, 21, 30).

Along with the altered myofibrillar structure, we find fragmented and out-of-register internal membranes involved in myofiber excitation and Ca^2+^ handling. Despite these changes, we observed a small yet significant gain in the magnitude of Ca^2+^ release accompanied by reduced Ca^2+^ kinetics. Together, these changes do not align with the significant reduction in contractile force in the KI muscles but may contribute to the identified alterations in contractile kinetics. Given our identification of disrupted sarcomere structure, it is possible that deficits in lateral force transmission across myofibrils contribute to the significant reduction in contractile force in the KI muscles. Such deficits would be additive to the identified deficits in Ca^2+^ sensitivity at the cross-bridge level and would occur independently of a change in tension redevelopment, which we identified here. Along these lines, we and others have implicated similar structural alterations in dystrophic and aging muscles with an increased susceptibility to contraction-induced damage that predisposes to atrophy (31). We therefore speculate that the decreased musculoskeletal size seen in the KI mouse and patients arises, in part, from these structural changes seen early in postnatal development.

While our work provides important mechanistic insights into muscle weakness in the heterozygous E248K mice, the mechanism of tremor generation in both the mice and the patients remains unclear. Tremors can arise from both neurogenic and myogenic foci, with heterozygous E248K carriers aligned with the latter. However, each are thought to result from oscillations within the servomechanism γ-loop associated with the stretch reflex (32). Central to this mechanism are innervating intrafusal muscle fibers whose activation by the magnitude and rate of change in muscle length regulates the reflex arc (33). Evidence that the observed 10 Hz tremor in the human heterozygous E248K patients is similar to the reports of postural tremor frequency of 8–12 Hz in adults (34) supports all tremor foci acting through this common pathway. Within this context and given that sMyBP-C expression is restricted to the muscle sarcomere, our results suggest that aberrant contractile behavior within a muscle (i.e., fascicles, motor-units) and between functional muscle synergists induces oscillations within the reflex arc to generate tremors. This concept is further supported by evidence in the human patients that CNS adaptation of motor unit control decreases tremor occurrence in adulthood (13, 14) and by our findings in the E248K KI mouse model that the declining incidence of tremor with age can be unmasked by sedation to suppress CNS control.

While dysregulated contractile activation within the muscle is a parsimonious explanation for tremor initiation, other mechanisms may conspire with E248K or act independently to initiate tremor. In the heart, the genetic deletion of cMyBP-C initiates significant remodeling that predisposes to arrhythmia from a reduced expression of potassium channel subunits and systemic adrenergic activation (35). While such alterations in mutant muscles could alter excitability to promote contractile dysregulation and, thus, tremors, chronic β2 activation of intrafusal fibers has been implicated in the remodeling and altered activation of potassium channels to promote tremors outside of the muscle fiber per se (36). Our future studies will focus on systematically dissecting these possibilities to further understand the origin of the tremor.

It is unequivocal that the E248K pathogenic variant in sMyBP-C gave rise to these identified defects either directly or through remodeling secondary to the observed loss-of-function phenotype. While our studies are the first to formally describe (11) and mechanistically interrogate this potentially novel form of *MYBPC1* myopathy, dominant missense variants in additional sarcomeric genes, including *MYH2*, *MYH7*, *MYL2*, *TNNT1*, *TPM3*, and *NEB*, also result in myopathy with tremor in the absence of obvious neuropathy (recently reviewed in ref. 37). Our findings are, therefore, highly impactful in establishing not only a new form of *MYBPC1* myopathy, but of a myopathy encompassing several genes that likely originates from structurally disordered and dysregulated sarcomeres. Consideration of these findings will inform accurate disease diagnosis and will be essential for effective and targeted treatment design.

## Methods

### Generation of the E248K KI mouse model.

Generation of a KI mouse model containing the E249K substitution corresponding to the human E248K pathogenic variant (11) was performed by the Jackson Laboratory; to avoid confusion, we refer to the model as E248K KI model. Embryonic stem cells containing the desired pathogenic variant (c. 747 G>A) were generated with CRISPR/Cas9 technology using the following guide RNAs to target *MYBPC1* exon 8: sgRNA1, 5′ GCGATCTTCTCGTATTCGTT 3′, and sgRNA2, 5′ AGCGATCTTCTCGTATTCGT 3′. Validated clones were confirmed with the following primers set: forward (sense), 5′ TACTCTCCGGAAGTTTCCCAAG 3′, and reverse (antisense), 5′ TAGGATGCGACCACACACAC 3′. These validated clones were microinjected into blastocysts of C57BL/6J-Tyr^c-2J^ mice (https://www.jax.org/strain/000058) and then transferred into pseudopregnant females. Founder animals were identified by coat color and used for breeding to generate N1 mice as deliverables.

With the exception of body weight analysis presented in growth charts, data from female and male animals were combined, since both sexes appeared to be similarly affected, as shown in an inverted hang assay (Supplemental Figure 7; for description of the inverted hang test, please see below), which is in agreement with the sex-independent manifestation of the disease in human carriers (11).

### Survival curves.

Survival curves were generated for WT and heterozygous KI animals from P7 through 4 weeks of age. Overall survival was determined from birth through 6 months of age for both WT and heterozygous KI mice.

### Growth and weight charts.

WT and heterozygous KI mice were weighed daily starting at P21 through 12 weeks of age, followed by weekly weighing until they reached 24 weeks of age. Muscle/total body weight ratios were determined for heart, diaphragm, quadriceps, gastrocnemius, tibialis anterior, soleus, and flexor digitorum brevis muscles at 4 weeks.

### ddPCR.

RNA from 4-week-old WT and heterozygous KI EDL and soleus muscles was extracted with QIAzol (Qiagen, catalog 79306). After the RNA pellet was washed with ethanol, subsequent purification was completed using the RNeasy MinElute Cleanup Kit (Qiagen, catalog 74204). To obtain cDNA, reverse transcription was performed with 500 ng of extracted RNA and random hexamers using the SuperScript IV Reverse Transcriptase (Invitrogen, catalog 18090010). A custom TaqMan SNP genotyping assay was designed to recognize c. 747 G (WT) with a VIC-labeled probe or c.747 A (pathogenic variant) with a 6-FAM–labeled probe according to the following sequence: 5′ GGAGGTGAAGCAGCAGGAAGAGGAGCCTGAGATAGACGTGTGGGAGCTGCTGAAAAATGCCAACCCCAACGAATACGA[G/A]AAGATCGCTTTCCAGTATGGCATCACCGACTTGCGTGGCATGCTTAAGCGGCTCAAGCGCATGCGCAGGGTGGAAAAGAAGAGCGCAG 3′ (Thermo Fisher Scientific, catalog 4332077). The ddPCR assay was performed with a C1000 Touch Thermal Cycler (catalog 185-1197) after droplet generation with a Bio-Rad QX200 Automated Droplet Generator (catalog 186-4101) and a QX200 Droplet Reader (catalog 186-4003). A 2X Probe Supermix (No dUTP; catalog 186-3023) was used with 900 nM final primer concentration, 200 nM probe concentration, and a 1:10 dilution of cDNA as template. Droplet generation and thermocycling were completed according to the manufacturer’s instructions (Bio-Rad). QuantaSoft software was utilized for droplet analysis after setting an appropriate threshold across samples and normalizing to WT.

### Evaluation of tremor intensity/duration in neonatal pups.

To establish the link between tremor and pathology in our model, we profiled the duration and intensity of tremors in a time-sampling manner over a period of 10 minutes with 1-minute duration for each measurement (minute 1, 3, 5, 7, and 9) and 1-minute break between measurements (minutes 2, 4, 6, and 8), as described in ref. 38. Tremor intensity was evaluated using a 4-point ranked scale, as follows: 0, no tremor; 1, weak tremor in limited areas, such as forelimbs and hindlimbs; 2, moderate tremor in extended areas including limbs, upper body, trunk, and head; and 3, intense tremor over the whole body. Tremor evaluation was performed during postnatal development at P1, P3, P5, P7, P10, and P14 by 2 blinded individuals. Data were averaged for the 10-minute observation period for each genotype on each day of the study.

### Righting reflex test.

The locomotor abilities of neonates were evaluated using the righting reflex test as described on the Treat-NMD website (http://www.treat-nmd.eu/downloads/file/sops/md/MD_ M.2.2.002.pdf) with a cutoff time of 60 seconds as in ref. 39. This assay scores the pup’s ability to return to 4 paws after they are placed in a supine position. The test was repeated 3 times by 2 blinded individuals with at least 2-minute intervals between each test using neonates at P4, P6, and P8. The time needed to fully right (i.e., all 4 paws visibly on the ground) was recorded in seconds. If the mouse failed to invert after 60 seconds, the test was stopped and a maximum score of 60 was given. Data were averaged for each genotype on each day of the study.

### Evaluation of tremor intensity in young adult animals.

Tremor in young adult animals (4 weeks old) was assessed using a custom-designed tremor plate. After 90 seconds of 2% isoflurane exposure, anesthetized mice were placed into a GRASS instrument chamber cantilevered to an FT-03 force transducer. As animals recovered, vibrations were sampled at 1 kHz for 6 minutes using a GRASS A/D interface and GRASSlab software. Individual trials were postprocessed with FFT analysis in MATLAB software.

### Open field.

Mice were acclimated to the testing room in their home cage for 60 minutes and then placed into a voluntary activity enclosure equipped with horizontal and vertical infrared beams and a tracking camera. Horizontal movement and rearing activity were measured for 30 minutes with AnyMaze software (Stoelting Co.). Following testing, mice were returned to their home cage. Mice were tested at 4 and 6 weeks of age, and the test was repeated on 2 consecutive days. Data points correspond to averaged assay measurements for each animal at the indicated time points normalized to distance traveled.

### Ataxia box (parallel rods).

Mice were acclimated to the testing room in their home cage for 60 minutes, and then placed into an ataxia box (also referred to as parallel rods) testing chamber to test locomotion skills as in ref. 40. The testing chamber was equipped with parallel stainless steel rods of varying diameters (1.6 mm and 3.2 mm) and an interrod spacing of 4 or 8 mm from the edge of one rod to the edge of the next rod. A single rod oriented perpendicularly on each side connected the parallel rods. The floor was set on top of an acrylic plastic frame that separated it from a stainless steel plate set 1 cm below the rod floor. For testing, mice were placed in the center of the parallel rods and allowed to move about the chamber for 10 minutes and monitored by a tracking camera. Horizontal movement parameters were recorded, as well as the number of foot slips, using AnyMaze software. Measurements were repeated over 3 consecutive days using 4- and 6-week-old mice of both genotypes. Immobility was defined as 65% of the body exhibiting no movement for at least 2 seconds, and freezing was defined as no movement detected for at least 1 second. Data points correspond to averaged assay measurements for each animal at the indicated time points normalized to distance traveled.

### Inverted hang test.

Overall muscle strength/endurance was measured by an inverted hang test (4-limb hanging wire test). The animal was placed on a wire grid approximately 50 cm above a soft padded surface. The grid was slowly rotated over 2 seconds until the animal was fully inverted, at which point a timer was started, and time until the animal dropped was recorded. The mouse was returned to its cage and allowed to rest for at least 3 minutes. The assay was repeated 3 times in 1 day, and the average time on the grid was calculated. If the mouse did not fall, the test was stopped after 3 minutes, and a maximum time was recorded. Mice at 4, 6, and 8 weeks of age were evaluated. Data points correspond to averaged assay measurements for each animal at the indicated time points.

### Plethysmography.

Whole body plethysmography was used to assess respiratory flow in 2- and 4-week-old unrestrained, nonanesthetized mice. Mice were introduced into plethysmography chambers (Buxco Research Systems), and following exploration and grooming behaviors, mice settled and were studied during quiet rest. A 20-minute baseline recording was collected. Ventilation was determined during steady-state condition. PIF and peak expiratory flow (PEF) were measured and normalized for body mass; no statistical difference was observed in PEF measurements between WT and heterozygous KI mice.

### Radiography.

X-ray radiographs were performed on 6-month-old mice with a digital Faxitron radiography machine (Faxitron X-Ray). Curvature of the spine (kyphotic index) was determined as in ref. 41 by drawing 2 lines between the caudal margin of the seventh cervical vertebra and the caudal margin of the sixth lumbar vertebra.

### Antibodies.

Antibodies used for immunoblotting and immunofluorescence were as follows: rabbit polyclonal: actin (A2066, Sigma-Aldrich), Hsp90 (4874S, Cell Signaling Technologies), sMyBP-C (SAB3501005, Sigma-Aldrich), and fMyBP-C (PAB19214, Abnova); mouse monoclonal: myosin (fast skeletal; M1570, Sigma-Aldrich), and myosin (slow skeletal; clone NOQ7.5.4D, Sigma-Aldrich).

### Generation of protein lysates and immunoblotting.

Freshly isolated diaphragm, EDL, and soleus muscles were collected from 2- and 4-week-old mice and flash frozen in liquid nitrogen. Diaphragm lysates were prepared with Qiagen TissueLyser LT for 2 minutes at 50 Hz in modified NP-40 lysis buffer (in mM: 10 NaPO_4_ [pH 7.2], 2 EDTA, 10 NaN_3_, 120 NaCl, 0.5% deoxycholate, and 0.5% NP-40) supplemented with complete protease inhibitors (Roche). EDL and soleus lysates were prepared in the same lysis buffer using a hand homogenizer and vortexed every 15 minutes for 2 hours on ice. Following addition of Nupage LDS sample buffer and reducing agent and boiling at 95°C for 5 minutes, 20 μg of diaphragm, EDL, or soleus protein lysates were fractionated by 4%–12% SDS-PAGE and transferred to nitrocellulose, which was cut into strips that were probed with the indicated primary antibodies, followed by the appropriate alkaline phosphatase-conjugated secondary antibodies (Jackson ImmunoResearch). Immunoreactive bands were visualized with the Tropix chemiluminescence detection kit (Thermo Fisher Scientific). In the fast-twitch EDL muscle, myosin slow levels could not be reliably detected. Moreover, depending on the muscle, 2 bands were occasionally detected for fMyBP-C with molecular weights of ~130 kDa and 145–150 kDa. The band detected around ~130 kDa is shown and quantified, as it is closest in molecular weight to the reported size of fMyBP-C. There is a possibility that the band detected around 145–150 kDa is nonspecific, although it should not be dismissed that there may be unreported, larger fMyBP-C variants, as is the case for sMyBP-C. Densitometry was performed with ImageJ software (NIH). Individual points on graphs represent biological samples; of note, some blots were flipped horizontally for presentation purposes due to sample order during loading. Moreover, due to the similar molecular weights of slow and fast myosin, as well as sMyBP-C and fMyBP-C, it was not feasible to probe for all 4 proteins in the same blot. Accordingly, representative immunoblots are shown for each protein of interest (i.e., sMyBP-C, slow myosin, fast myosin, fMyBP-C, and actin) and the loading control (i.e., Hsp90), while panels are separated by white spaces to indicate different gels or strips obtained from the same gel; Supplemental Figure 8, which includes the unedited blots, is provided with representative blots marked with color-coded boxes and additional proteins denoted in black font.

### H&E staining.

Frozen sections (10 μm thickness) of 2- and 4-week-old WT and heterozygous KI EDL and soleus muscles were stained with H&E, according to the manufacturer’s instructions (Abcam, catalog ab245880) and mounted with Permount (Thermo Fisher Scientific, catalog SP15-100). Specimens were analyzed under a Nikon Eclipse Ti2 inverted microscope equipped with 4× and 10× objective lenses.

### Immunofluorescence staining and confocal microscopy.

Two- and 4-week-old mice were deeply anesthetized and euthanized by exsanguination, and EDL and soleus muscles were rapidly harvested, embedded in OCT (VWR), and frozen. Muscles were cryosectioned (12 μm thickness), permeabilized with 0.1% Triton-X for 20 minutes, and blocked in PBS containing 2% BSA, 1 mM NaN_3_, and 2% goat serum for 1 hour at room temperature before immunolabeling with the indicated primary antibodies diluted in PBS/BSA/NaN_3_ overnight at 4°C. Samples were washed with PBS containing 0.1% Tween 20, counterstained with secondary antibodies conjugated with Alexa Fluor 488 or Alexa Fluor 568 in PBS/BSA, followed by another wash with PBS/0.1% Tween 20 and mounted with ProLong Diamond Antifade Mountant (Thermo Fisher Scientific). Specimens were analyzed under a Nikon Eclipse Ti2 spinning disk confocal microscope equipped with a 60×, 1.49 numerical aperture oil immersion TIRF objective using the same dual laser settings with 516.5 and 600.5 nm emission wavelengths, pinhole size of 50.00 μm, and 500 ms exposure time.

### CSA determination and fiber typing.

Frozen sections (10 μm thickness) of 4-week-old EDL muscles were blocked with M.O.M. (Vector Laboratories, catalog MKB-2213) for 1 hour at room temperature. Sections were incubated with the following antibodies from DSHB: myosin heavy chain type I/IgG2b (catalog BA-D5), myosin heavy chain type IIA/IgG1 (catalog SC-71), and myosin heavy chain type IIB/IgM (catalog BF-F3) at 1:100 dilution, and laminin (MilliporeSigma, L9393) at 1:30 dilution for 45 minutes at 37°C in PBS-1% BSA. After washing with PBS, secondary antibodies (anti–mouse IgG2b Alexa 405, –IgG1 Alexa 488, –IgM Alexa 594, and anti–rabbit IgG Alexa 647) were added for 30–40 minutes at 37°C at 1:200 dilution (Jackson ImmunoResearch). Following washing with PBS, slides were mounted with ProLong Diamond Anti-fade Mounting Medium (Thermo Fisher Scientific, P36970). NIS-Elements Viewer software was used to trace cell boundaries, determine CSA, and analyze fiber type staining. A total of 33,635 WT and 23,362 heterozygous KI fibers were analyzed.

### Ex vivo contractility studies.

Muscle performance was assessed ex vivo using methods previously described (42). Briefly, 4-week-old EDL muscles were dissected and secured at each tendon with silk suture (5-0), and diaphragm strips were dissected with rib and central tendons attached. Each muscle was mounted in an in vitro bath between a fixed post and force transducer (300B-LR; Aurora Scientific) operated in isometric mode. Muscles were maintained in physiological saline solution (pH 7.6) containing (in mM) 119 NaCl, 5 KCl, 1 MgSO_4_, 5 NaHCO_3_, 1.25 CaCl_2_, 1 KH_2_PO_4_, 10 HEPES, and 10 glucose and maintained at 30°C under aeration with 95% O_2_ and 5% CO_2_ throughout the experiment. Resting tension and stimulation current were iteratively adjusted for each muscle to obtain optimal twitch force. During a 5-minute equilibration period, single twitches were elicited at every 30 seconds with electrical pulses (0.2 ms) via platinum electrodes running parallel to the muscle. Optimal resting tension was determined, and isometric tension was evaluated by 250 ms trains of pulses delivered at 1, 10, 20, 40, 60, 80, 100, 150, 250, and 300 Hz.

To normalize force production to muscle mass, at the end of the above experimental protocol, the muscle rested for 5 minutes, at which time muscle length was determined with a digital micrometer. Subsequently, muscle was trimmed proximal to the suture connections, blotted, and weighed. The CSA (μm^2^) for each EDL muscle was determined by dividing the mass of the muscle (g) by the product of its length (*L*_o_, μm) and the density of each muscle (1.06 g/cm^3^) (43). Muscle output was then expressed as isometric tension (mN/mm^2^) determined by dividing the tension (mN) by the CSA (μm^2^).

### Ca^2+^ signaling in intact EDL muscle.

EDL muscles were dissected from 4-week-old WT and heterozygous KI mice. Distal and proximal tendons were tied using suture silk (5-0). Muscles were mounted in custom fabricated glass-bottomed chamber (Four-Hour Day Foundation) bathed in HEPES-buffered Ringer solution containing (mM) 140 NaCl, 4 KCl, 1 MgSO_4_, 5 NaHCO_3_, 10 glucose, and 10 HEPES (pH 7.4) at room temperature. To avoid motion artifacts, the Ringer solution was supplemented with Myosin II ATPase inhibitor N-benzyl-p-toluene sulfonamide (BTS; 50 μM). Experiments were performed on an inverted Nikon C2 confocal microscope (Nikon Instruments) using a 20× 0.75 NA objective. Ca^2+^ transients of individual fibers within the EDL muscle were evaluated after loading the muscle with 5 μM Fluo4-AM (45-minute incubation) followed by 10-minute deesterification. The Ca^2+^ sensor was excited using a 488 nm laser, and emission was collected at 510–550 nm using fast line scan mode (1.8 ms per line) for 5 seconds. To initiate Ca^2+^ signaling, EDL muscles were electrically stimulated at 40 Hz (500 μs, square pulses) for 0.5 seconds. The fluorescence intensity profile for each fiber was obtained using ImageJ (NIH), and Ca^2+^ signaling parameters were calculated using a home-made Python script.

### Skinned single-fiber mechanical measurements.

EDL muscles were dissected from 4-week-old mice, tied to toothpicks, and placed in a 1:1 ratio of glycerol and relaxing solution containing (in mM) 10 EGTA (96.5% pure), 100 N,N-bis(2-hydroxyethyl)-2-aminoethanesulfonic acid (BES), 6.87 MgCl2, 31 Kprop, 5 NaN_3_, 10 phosphoenolpyruvate (PEP), 1 DTT, and 5.83 ATP (99% pure) adjusted to pH 7 with KOH for a minimum of 3 and a maximum of 14 days. On the day of experimentation, single fibers were gently pulled from the fiber bundle, as in refs. 24 and 25. The experimental apparatus was mounted on the stage of an inverted microscope (model IX70; Olympus Instrument Co.), which was placed on a pneumatic vibration isolation table. Mechanical measurements were performed using a capacitance gauge force transducer (Model 403, sensitivity of 20 mV/mg and resonant frequency of 600 Hz; Aurora Scientific). Length changes were presented to one end of the fiber via a DC torque motor (model 308c; Aurora Scientific) driven by voltage commands from a personal computer via a 16-bit D/A converter (AT-MIO-16E-1; National Instruments Corp.). Fibers were attached between the force transducer and length motor by placing the ends of the fiber into stainless steel troughs (25 gauge). The fiber ends were secured by overlaying a ~0.5 mm length of 3-0 monofilament suture (Ethicon). The suture secured the fiber into the troughs by tightening 2 loops of 10-0 monofilament (Ethicon) at each end. The attachment procedure was performed under a stereomicroscope (90× zoom). Force and length signals were digitized at 1 kHz and stored on a personal computer using Lab-View for Windows (National Instruments Corp.). Simultaneous sarcomere length measurements of force and length were obtained via the IonOptix SarcLen system, which used a FFT algorithm of the video image of the fiber.

All mechanical measurements on skeletal muscle fibers were performed at 15°C ± 1°C. Following attachment, the relaxed preparation was adjusted to a sarcomere length slightly above slack length by manual manipulation of the length micrometer on fiber mount. For tension-pCa relationships, the preparation was first transferred into pCa 4.5 solution for maximal activation and subsequently transferred into a series of submaximal activating pCa solutions, ending back in pCa 4.5 maximal activating solution. At each pCa, steady-state tension was allowed to develop, and the cell was rapidly slackened to determine total tension, after which the preparation was restretched to a value slightly greater than the original muscle length for ~2 ms and then returned to original muscle length. The fibers underwent approximately 3 slack-restretch maneuvers at each Ca^2+^ activation level. Tensions in submaximal activating solutions were expressed as a fraction of tension obtained during maximal Ca^2+^ activation. The maximal tension value used to normalize submaximal tensions was obtained by averaging maximal activation at the beginning and end of the protocol. Force development following a slack-restretch maneuver was fit by a single exponential equation.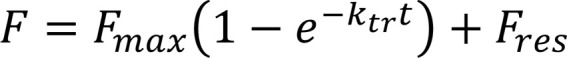, where *F* is force at time *t*, *F_max_* is maximal force, *k_tr_* is the rate constant of force development, and *F_res_* represents residual tension immediately after the slack-restretch maneuver.

Force-velocity and power-load measurements were evaluated by performing a series of subisometric force clamps, as previously described (24) at 15°C ± 1°C. Skeletal muscle fibers were placed in activating solution, and once steady-state force was developed, a series of subisometric force clamps was performed to determine isotonic shortening velocities. Using a servo-system, force was maintained constant for a designated period of time (150–250 msec) while the length change was continuously monitored. Following the force clamp, the fiber was slackened to reduce force to near zero to allow estimation of the relative load sustained during isotonic shortening. The muscle fiber preparations were kept in maximal Ca^2+^-activating solution for 3–4 minutes, during which 10–15 force clamps were performed without significant loss of force. Skeletal muscle fiber length traces during loaded shortening were fit to a single decaying exponential equation: *L* = *Ae^–kt^* + *C*, where *L* is cell length at time *t*, *A* and *C* are constants with dimensions of length, and *k* is the rate constant of shortening. Velocity of shortening at any given time *t*, was determined as the slope of the tangent to the fitted curve at that time point. Velocities of shortening were calculated by extrapolation of the fitted curve to the onset of the force clamp (i.e., *t*=0). Hyperbolic force-velocity curves were fit to the relative force velocity data using the Hill equation: (*P* + *a*)(*V* + *b*) = (*P*_0 _+ *a*)*b*, where *P* is force during shortening at velocity *V*, *P*_0_ is the peak isometric force, and *a* and *b* are constants with dimensions of force and velocity, respectively. Power-load curves were obtained by multiplying force × velocity at each load on the force-velocity curve.

### Electron microscopy and FFT analysis.

Soleus, EDL and diaphragm muscles from 4-week-old mice were fixed in 2% paraformaldehyde, 2.5% glutaraldehyde, and 0.1M PIPES buffer (pH 7.4), washed with 0.1M PIPES buffer, and postfixed with 1% osmium tetroxide/1.5% potassium ferrocyanide in 0.1M PIPES buffer for 1 hour at 4°C. Specimens were subsequently treated with 1% tannic acid in H_2_0 for 15 minutes, followed by en bloc staining with 1% (w/v) uranyl acetate and dehydration using 30%, 50%, 70%, 90%, and 100% ethanol in a series. Following dehydration, samples were infiltrated and embedded in Araldite-Epoxy resin (Araldite, EMbed 812; Electron Microscopy Sciences) according to the manufacturer’s recommendations. Ultrathin sections at ~70 nm thickness were cut on a Leica UC6 ultramicrotome (Leica Microsystems Inc.) and examined under a Tecnai T12 transmission electron microscope (Thermo Fisher Scientific) operated at 80 kV. Images were acquired with an AMT bottom-mount CCD camera and AMT600 software (Advanced Microscopy Techniques), and exposure was digitally adjusted.

For quantitative analysis of myosin filament CSA and interfilament distance, electron micrographs obtained from 2- and 4-week-old WT and heterozygous KI muscles were blindly analyzed by a custom MATLAB script, which is deposited in a public database (https://github.com/RunYangYang/Sarcomeric-deficits-underlie-MYBPC1-associated-myopathy-with-myogenic-tremor.git, commit ID feba32a185c0f5cb05d9761868c1a1807080aaf2). Thick myosin filaments were identified by image segmentation involving median blurring, binarization/thresholding/closing, 2D convolution, and subsequent Canny edge detection. The CSA of individual myofilaments was obtained by filling the closed contours, measuring the sum of pixels, and multiplying with the scale bar indication. The distance between adjacent myofilaments was calculated by measuring the average distance between the mass centers of the 6 closest neighboring myofilaments and subtracting the average diameter of the myofilaments by approximating a round shape with the same area within each muscle genotype. Two electron micrographs were analyzed per muscle by quantifying 3 randomly selected regions per micrograph including > 100 myofilaments.

Moreover, the number of animals per genotype that demonstrated Z-disc streaming was quantified to determine the percent occurrence of the phenotype. Specifically, at 2 weeks, 2–7 images per animal were evaluated: diaphragm, *n* = 3 WT (1 male and 2 female) and *n* = 2 heterozygous KI (2 female); EDL, *n* = 4 WT (2 male and 2 female) and *n* = 3 heterozygous KI (1 male & 2 female); and soleus, *n* = 2 WT (female) and *n* = 2 heterozygous KI (2 female). Similarly, at 4 weeks, 5–8 images per animal were analyzed: diaphragm, *n* = 4 WT (2 male and 2 female) and *n* = 4 heterozygous KI (2 male & 2 female); EDL, *n* = 4 WT (2 male and 2 female) and *n* = 4 heterozygous KI (2 male & 2 female); and soleus, *n* = 3 WT (2 male and 1 female) and *n* = 3 heterozygous KI (2 male and1 female).

Lastly, for quantitative analysis of the area occupied by mitochondria, each organelle was manually delineated with the freehand selection tool in FIJI, and area was measured. Three and 5 mitochondria were analyzed per image at 2 and 4 weeks, respectively, and 3 images were evaluated per animal; reported values correspond to averaged measurements.

### Statistics.

Statistical tests, sample numbers, and number of biological/technical repeats are provided in figure legends. The following tests were utilized depending on the data: 1-tailed Student’s *t* test, 2-tailed Student’s *t* test, repeated-measures 1-way ANOVA, 2-way ANOVA, Mann-Whitney *U* test, Kruskal-Wallis test, or 2-tailed unpaired *t* test. Values are expressed as mean ± SEM; ^#^*P* < 0.05, *P < 0.01, ****P < 0.0001.

### Study approval.

All animal work was conducted under protocols approved by the IACUC of the University of Maryland School of Medicine using isogenic, age-matched WT and heterozygous E248K KI C57BL/6J mice. Animals are maintained with regular backcrossing to avoid off-target effects.

## Author contributions

Conceptualization was contributed by JGH, J Stavusis, CW, and AKK. Data curation was contributed by JGH, J Stavusis, HCJ, JCR, LH, and KSM. Formal analysis was contributed by JGH, J Stavusis, HCJ, JCR, LH, RZ, and CW. Funding acquisition was contributed by KSM, CW, and AKK. Investigation was contributed by JGH, J Stavusis, JCR, LH, JV, KSM, CW, and AKK. Methodology was contributed by JGH, J Stavusis, HCJ, KSM, CW, and AKK. Project administration was contributed by JGH, KSM, CW, and AKK. Resources were contributed by J Stains, KSM, CW, and AKK. Software was contributed by HCJ. Supervision was contributed by JGH, J Stains, KSM, CW, and AKK. Validation was contributed by JGH, J Stavusis, JCR, LH, KSM, CW, and AKK. Visualization was contributed by JGH, J Stavusis, JCR, LH, CW, and AKK. Writing of the original draft was contributed by JGH. Review and editing of the manuscript was contributed by JGH, J Stavusis, HCJ, JCR, LH, J Stains, KK, KSM, CW, and AKK.

## Supplementary Material

Supplemental data

Supplemental video 1

Supplemental video 2

Supplemental video 3

Supplemental video 4

Supplemental video 5

Supplemental video 6

Supplemental video 7

Supplemental video 8

Supplemental video 9

Supplemental video 10

Supplemental video 11

Supplemental video 12

Supplemental video 13

Supplemental video 14

Supplemental video 15

Supplemental video 16

## Version 1 08/26/2021

In-Press Preview

## Version 2 10/08/2021

Electronic publication
