# Supplementary material for: Sarcomeric deficits underlie MYBPC1-associated myopathy with myogenic tremor
Source: JCI Insight. 2021 Oct 8;6(19):e147612. doi: 10.1172/jci.insight.147612 (PMC8525646; doi:10.1172/jci.insight.147612)
Supplement: Supplemental data [file jciinsight-6-147612-s145.pdf]

Supplemental Information

Supplemental Table 1. Body weight and muscle measurements in 4-week old mice.

	**WT (n=16)**		**KI (n=14)**	
	**Weight**	**Muscle/Body Weight Ratio (mg/g)**	**Weight^**	**Muscle/Body Weight Ratio^^ (mg/g)**
Body (g)	15.36 ± 0.72	-	10.77 ± 0.85*	-
Heart (mg)	76.33 ± 3.18	5.01 ± 0.13	57.77 ± 3.45*	5.53 ± 0.24
Diaphragm (mg)	50.44 ± 2.70	3.33 ± 0.18	41.19 ± 3.01#	3.94 ± 0.30
TA (mg)	21.44 ± 1.58	1.37 ± 0.05	16.95 ± 1.47*	1.55 ± 0.06#
Soleus (mg)	3.67 ± 0.20	0.24 ± 0.01	2.53 ± 0.23*	0.24 ± 0.01
Gastrocnemius (mg)	46.06 ± 4.12	2.91 ± 0.17	34.54 ± 3.74*	3.07 ± 0.17
Quadricep (mg)	45.47 ± 2.93	2.95 ± 0.13	38.18 ± 3.23#	3.48 ± 0.01#
FDB (mg)	2.39 ± 0.18	0.16 ± 0.01	1.97 ± 0.17#	0.20 ± 0.01

n=16 WT (8 male & 8 female) and n=14 heterozygous KI (6 male & 8 female); statistical significance was determined with Student's t-test, and values are expressed as average ± SEM. ^ compared to average WT body weight and muscle weights, ^^ compared to average WT Muscle/Body weight ratios, #<0.05, *<0.01.

Supplemental Figure 1

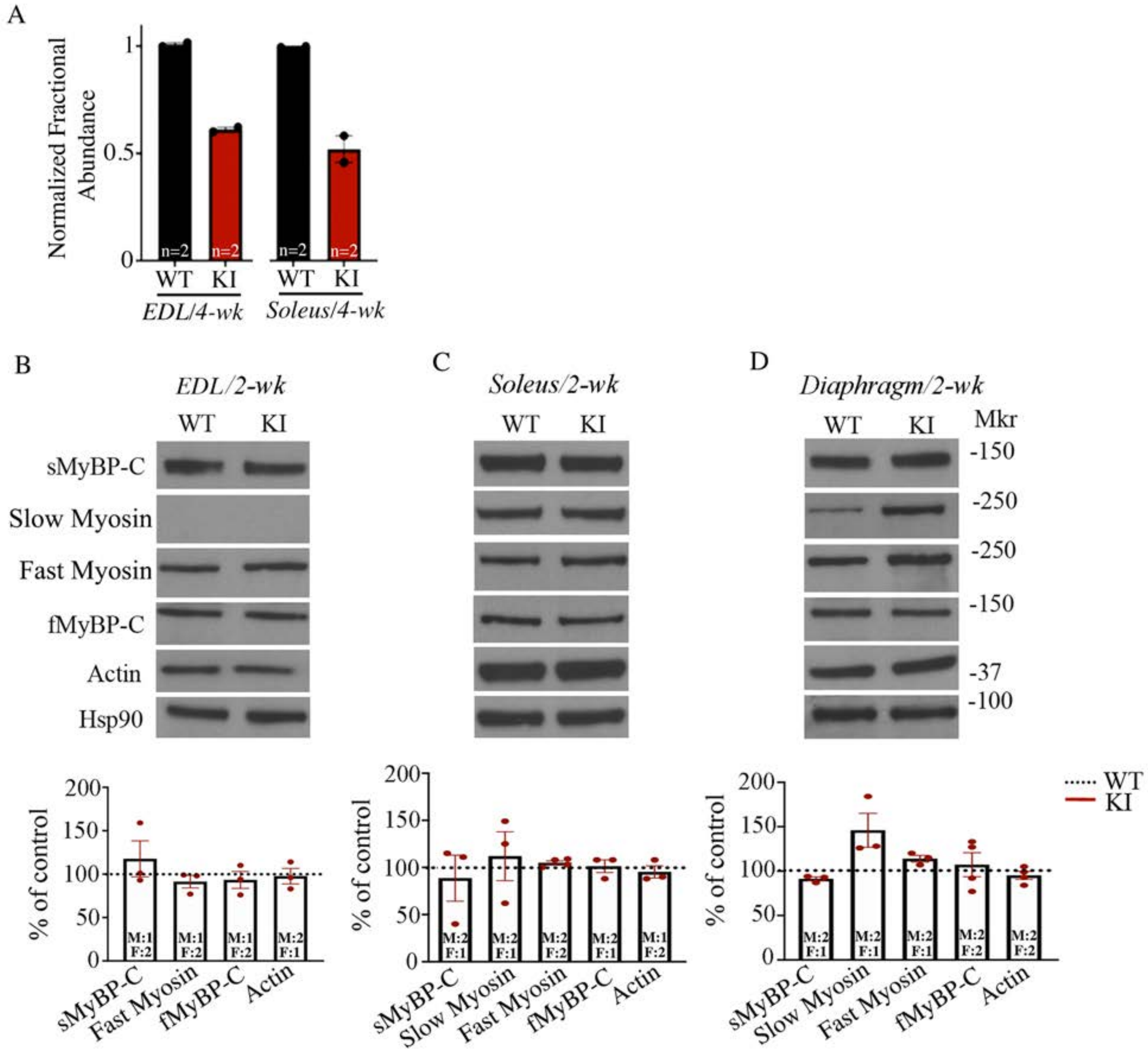

Supplemental Figure 1: Relative expression of WT and variant alleles at 4 weeks and biochemical analysis of heterozygous KI muscles at 2 weeks. (A) Droplet digital PCR (ddPCR) analysis using 4-week WT and heterozygous KI EDL and soleus muscles indicated a normalized fractional abundance value of 1 for WT and ~0.5 for KI muscles; for EDL, n=2 WT (2 male) and n=2 heterozygous KI (1 male & 1 female); for soleus, n=2 WT (1 male & 1 female) and n=2 heterozygous KI (1 male & 1 female). **(B-D)** Representative immunoblots and quantification

of the percent (%) expression of major myofibrillar proteins in heterozygous KI EDL **(B)**, soleus **(C),** and diaphragm **(D)** muscles indicated statistically unaltered protein expression at 2 weeks of age. All values are compared to WT, which was set to 100% following normalization to Hsp90 that was used as loading control. All blots were processed in parallel. Individual data points represent the number of biological samples evaluated for each protein obtained from both male (M) and female (F) mice, as indicated in the dot plots. Statistical significance was calculated with a normalized two-tailed Student's t-test.

Supplemental Figure 2

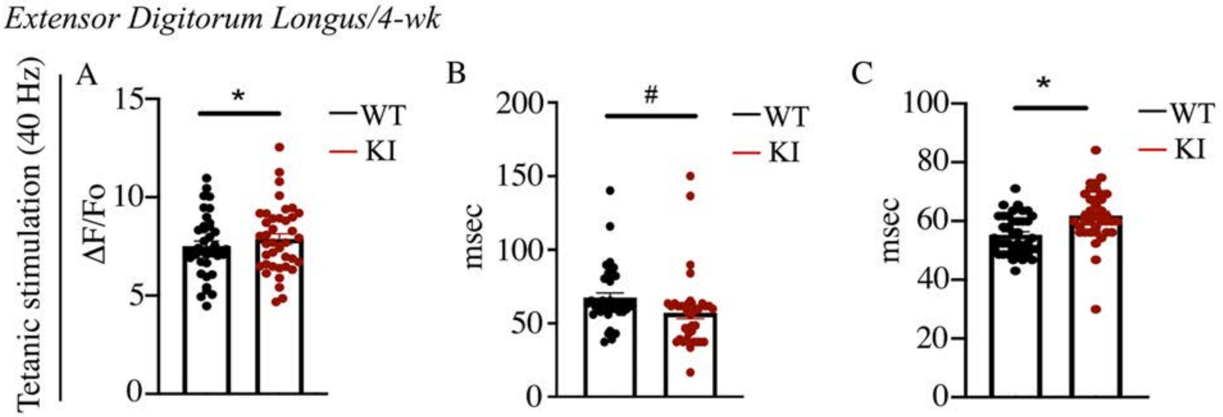

Supplemental Figure 2: Evaluation of Ca^{2+} cycling kinetics in intact heterozygous EDL muscles at 4 weeks. (A-C) Isolated EDL muscles from heterozygous KI animals exhibited significantly increased Ca^{2+} amplitude **(A)** after tetanic stimulation at 40 Hz compared to WT muscles; accompanied by significantly reduced Ca^{2+} rise time **(B)** and prolonged Ca^{2+} decay time **(C);** n=4 WT (4 male) and n=3 heterozygous KI (3 male). Statistical significance was calculated with two-tailed Student's t-test.

Supplemental Figure 3

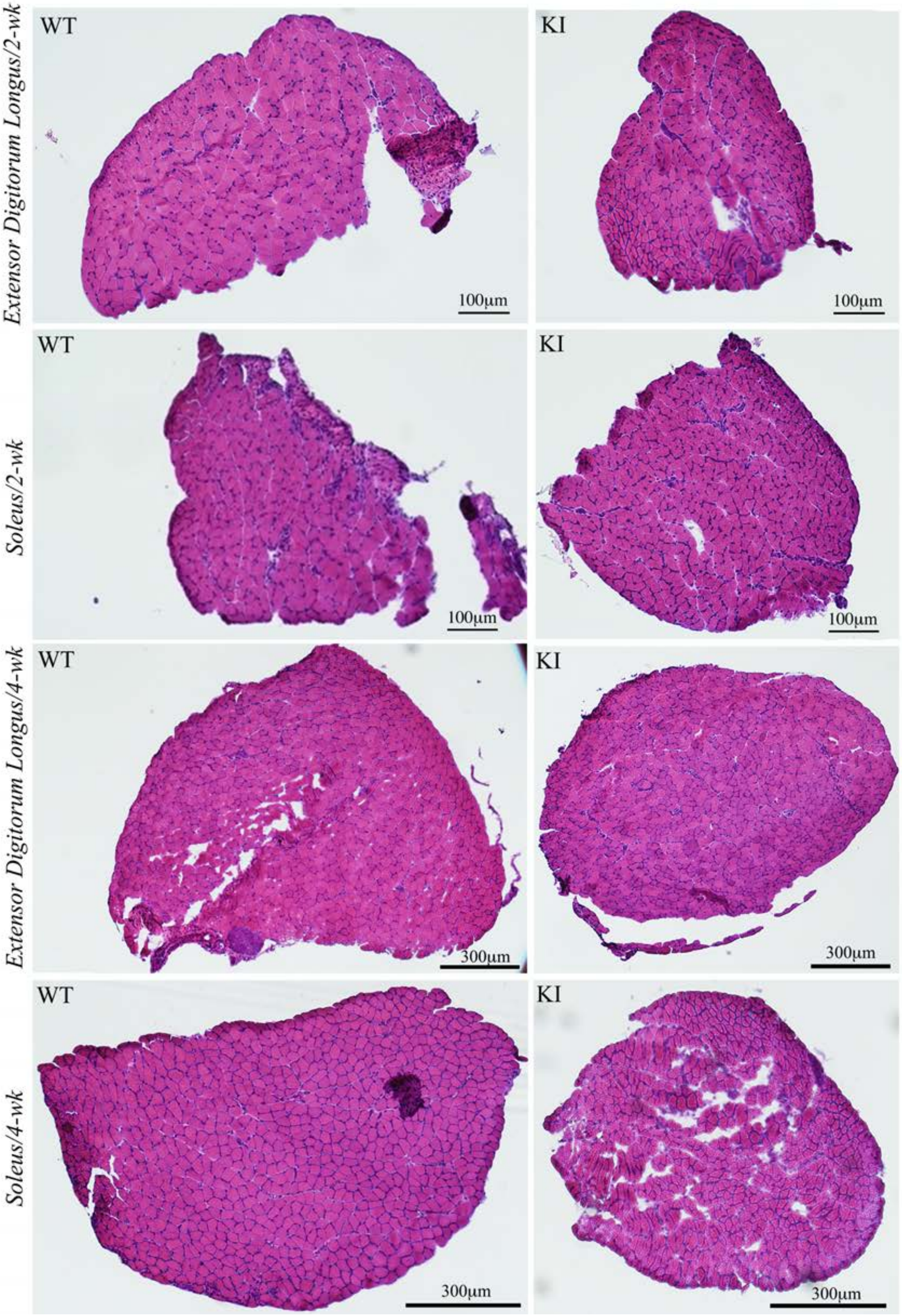

Supplemental Figure 3: Representative images of Hematoxylin & Eosin (H&E) stained sections of 2- and 4-week WT and heterozygous KI EDL and soleus muscles. No gross morphological alterations were observed between WT and KI EDL and soleus muscles either at 2 or 4 weeks of age; 2 weeks for both muscles: n=2 WT (2 female) and n=2 heterozygous KI (1 male & 1 female); 4 weeks for both muscles: n=2 WT (2 male) and n=2 heterozygous KI (2 male).

Supplemental Figure 4

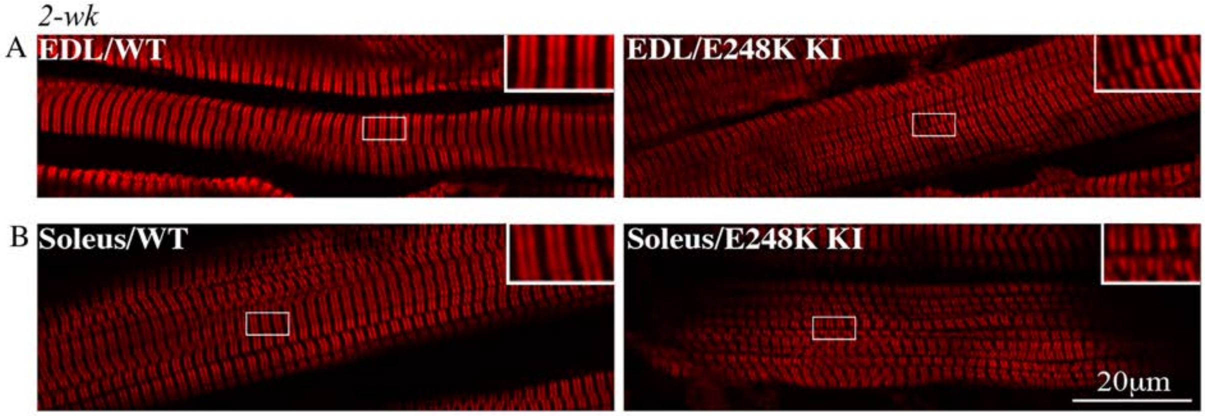

Supplemental Figure 4: Immunofluorescent analysis of heterozygous KI muscles at 2 weeks. (A-B) sMyBP-C assumed its typical distribution at the C-zone of A-bands in both WT and heterozygous KI EDL **(A)** and soleus **(B)** muscles, however bundles of split myofibrils and out-of-register sarcomeres were observed in heterozygous KI muscles; n=4 WT (2 male & 2 female) and n=4 heterozygous KI (2 male & 2 female).

Supplemental Figure 5

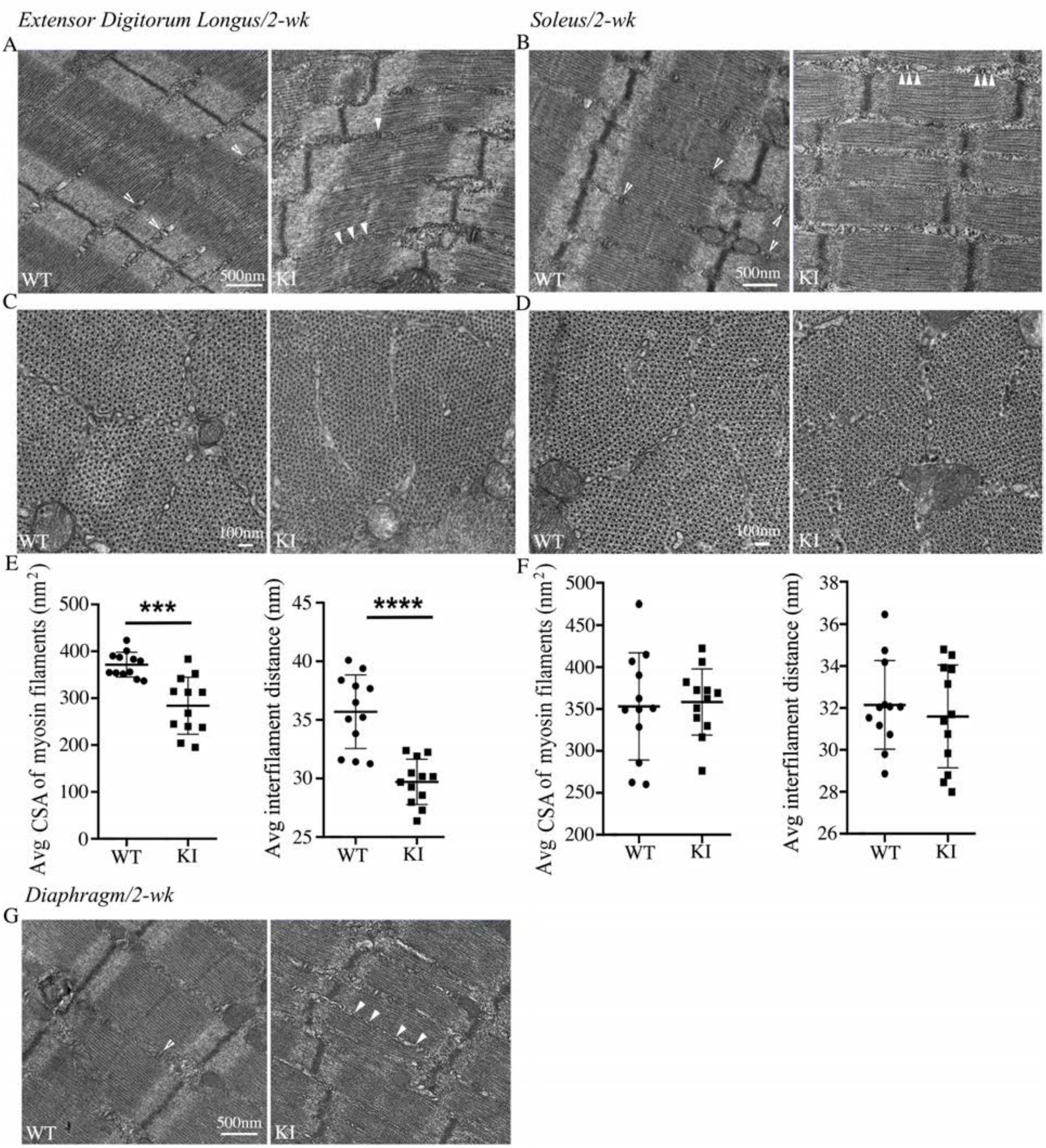

Supplemental Figure 5: Structural evaluation of heterozygous KI muscles at 2 weeks. (A-B) Electron micrographs of longitudinal sections of EDL **(A)** and soleus **(B)** muscles. WT muscles displayed properly organized and aligned sarcomeres with intact internal membranes forming

typical triads (open arrowheads) over A/I junctions. In contrast, heterozygous KI muscles exhibited less compact Z-discs and frequent Z-disc streaming, poorly defined A-, I- and M-bands, and fragmented and/or misaligned internal membranes (closed arrowheads); n=4 WT (2 male & 2 female) and n=4 heterozygous KI (2 male & 2 female). **(C-D)** Cross-sectional electron micrographs of WT and heterozygous KI EDL (C) and soleus (D) muscles indicated that both genotypes displayed normal hexagonal arrays of myosin filaments. **(E)** Both the CSA of individual thick filaments and the interfilament distance of heterozygous KI EDL muscles were significantly reduced compared to WT, as indicated by FFT analysis; n=2 WT (2 female) and n=2 heterozygous KI (2 female), two electron micrographs were analyzed per muscle by quantifying three randomly selected regions per micrograph including >100 myofilaments. **(F)** Contrary to KI EDL muscles, neither the CSA of individual thick filaments not the interfilament distance of heterozygous KI soleus muscles were significantly altered compared to WT; n=2 WT (2 female) and n=2 heterozygous KI (1 male & 1 female), two electron micrographs were analyzed per muscle by quantifying three randomly selected regions per micrograph including >100 myofilaments. **(G)** Electron micrographs of longitudinal sections of diaphragm muscles demonstrated similar structural alterations in heterozygous KI muscles as those observed in heterozygous KI EDL and soleus muscles; n=4 WT (2 male & 2 female) and n=4 heterozygous KI (2 male & 2 female). Statistical evaluation was performed with unpaired t-test (E-F).

Supplemental Figure 6

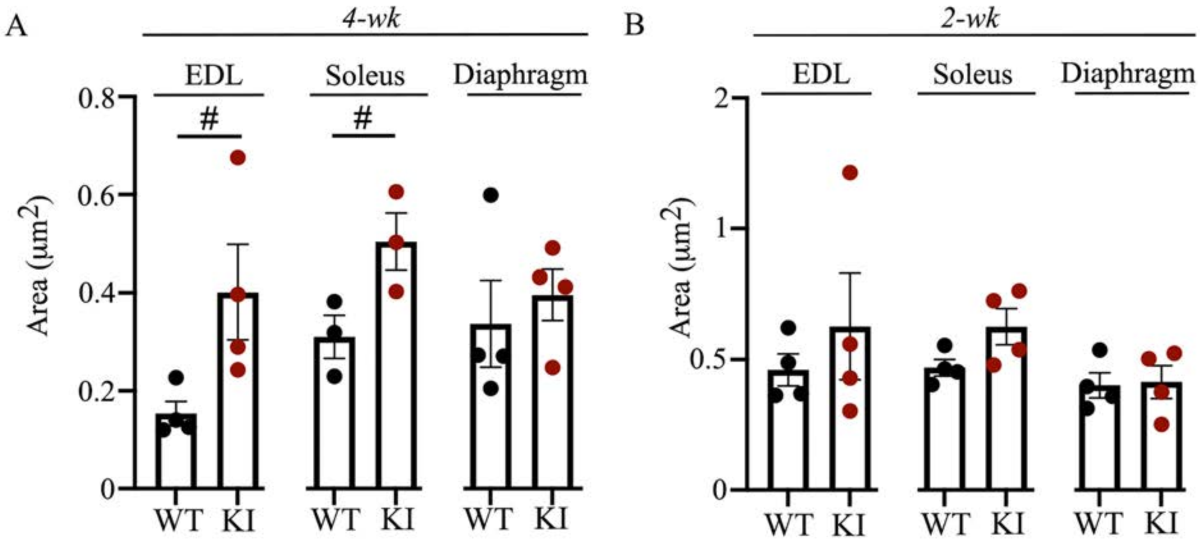

Supplemental Figure 6: Quantification of mitochondrial area in 2- and 4-week heterozygous KI muscles. (A) At 4 weeks, mitochondrial area was significantly increased in KI EDL and soleus muscles compared to WT, and trended similarly in KI diaphragm muscle. **(B)** At 2 weeks, mitochondrial area showed an increasing trend in KI EDL and soleus muscles, while in KI diaphragm it was comparable to WT; 2 weeks: n=4 WT (2 male & 2 female) and n=4 heterozygous KI (2 male & 2 female) for all muscles; 4 weeks: n=4 WT (2 male & 2 female) and n=4 heterozygous KI (2 male &2 female) for EDL and diaphragm and n=3 WT (2 male & 1 female) and n=3 heterozygous KI (2 male & 1 female) for soleus. Statistical evaluation was performed with a one-tailed Student’s t-test.

Supplemental Figure 7

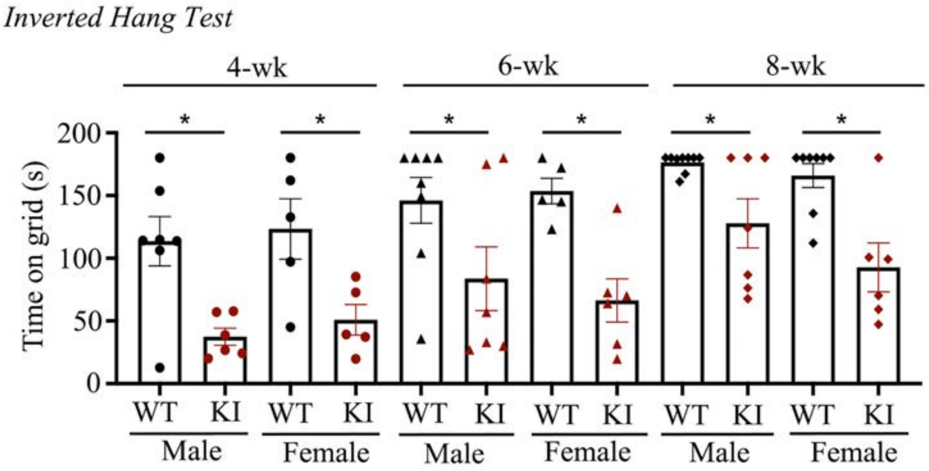

Supplemental Figure 7: Behavioral assessment of heterozygous E248 KI mice according to gender. The group of young adult heterozygous KI mice comprised of male and female animals that were evaluated in an inverted hang assay in figure 2 was divided according to gender and reanalyzed. Consistent with the absence of sex dimorphism in the human E248K carriers, heterozygous male and female E248K mice exhibited similar overall grip and body strength deficits at 4, 6 and 8 weeks of age; 4 weeks: n=12 WT (7 male & 5 female) and n=11 heterozygous KI (6 male & 5 female); 6 weeks: n=13 WT (8 male & 5 female) and n=13 heterozygous KI (7 male & 6 female); 8 weeks: n=17 WT (9 male & 8 female) and n=13 heterozygous KI (7 male & 6 female). Statistical significance was calculated with a two-tailed Student's t-test.

Supplemental Movie Titles and Legends

Supplemental Movie 1: Postnatal day 1 homozygous E248K KI pups exhibit severe head and body tremor and die within hours after birth.

Supplemental Movie 2: Postnatal day 1 wild-type littermates do not exhibit tremor and survive to adulthood.

Supplemental Movie 3: Postnatal day 1 heterozygous E248K KI pups exhibit intense limb tremor and to a lesser extent head and body tremor.

Supplemental Movie 4: Postnatal day 3 wild-type littermates do not exhibit tremor.

Supplemental Movie 5: Postnatal day 3 heterozygous E248K KI pups show intense tremor when stationary.

Supplemental Movie 6: Postnatal day 5 wild-type littermates do not exhibit tremor.

Supplemental Movie 7: Postnatal day 5 heterozygous E248K KI pups show intense tremor at rest or during locomotion.

Supplemental Movie 8: Postnatal day 7 wild-type littermates do not exhibit tremor.

Supplemental Movie 9: Postnatal day 7 heterozygous E248K KI pups exhibit intense tremor at rest and during action.

Supplemental Movie 10: Postnatal day 10 wild-type littermates do not exhibit tremor.

Supplemental Movie 11: Postnatal day 10 heterozygous E248K KI pups exhibit intense tremor at rest and during locomotion.

Supplemental Movie 12: Postnatal day 14 wild-type (right) and heterozygous E248K KI (left) pups moving freely around their cage; the wild-type pup is clearly larger compared to the KI pup, which exhibits tremor and delayed locomotion.

Supplemental Movie 13: A 6-week old wild-type male mouse shows stability in hindlimbs and forepaws during a hindlimb clasping assay.

Supplemental Movie 14: A 6-week old heterozygous E248K KI male mouse exhibits tremor in the hindlimbs and forepaws during a hindlimb clasping assay.

Supplemental Movie 15: An 8-week old wild-type male mouse is able to grab with its four paws, move, and stay on the grid for the entire experimental period (3min) during an inverted hang assay.

Supplemental Movie 16: An 8-week old heterozygous E248K KI male mouse fails to grab with its four paws, move or stay on the grid during experimentation in an inverted hang assay.

Supplemental Figure 8

2 weeks-EDL

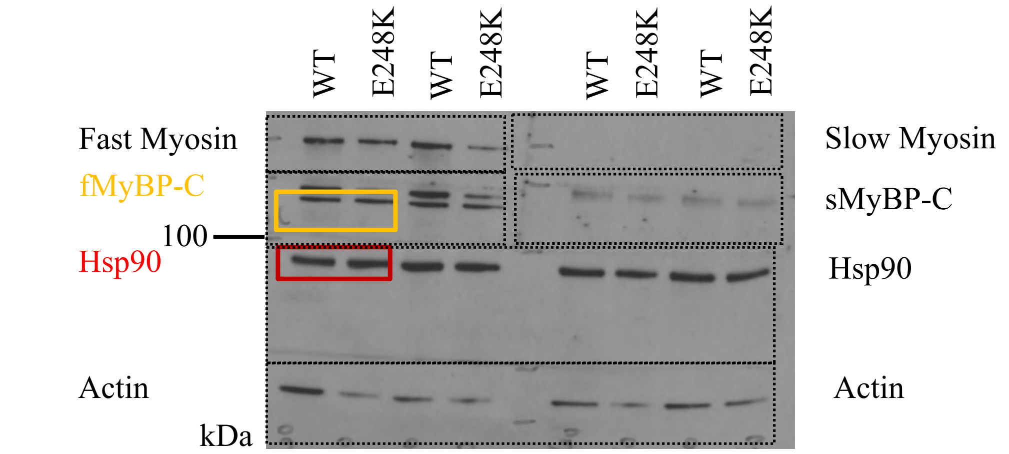

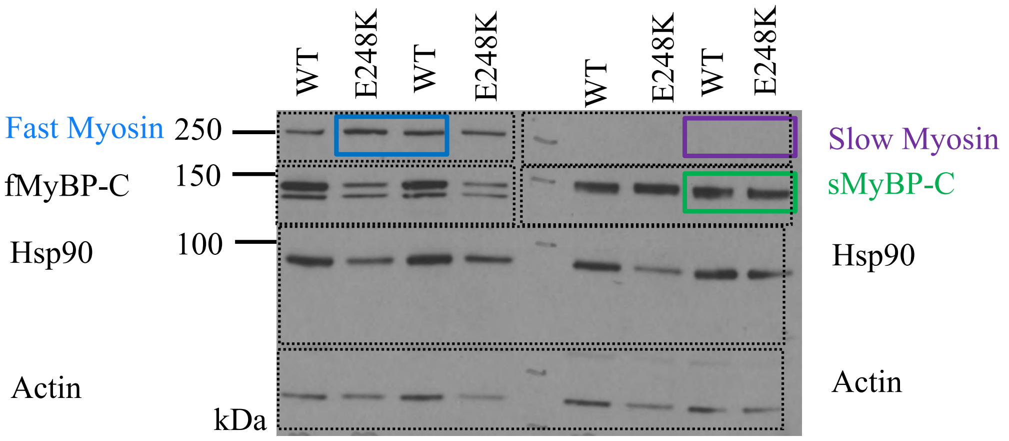

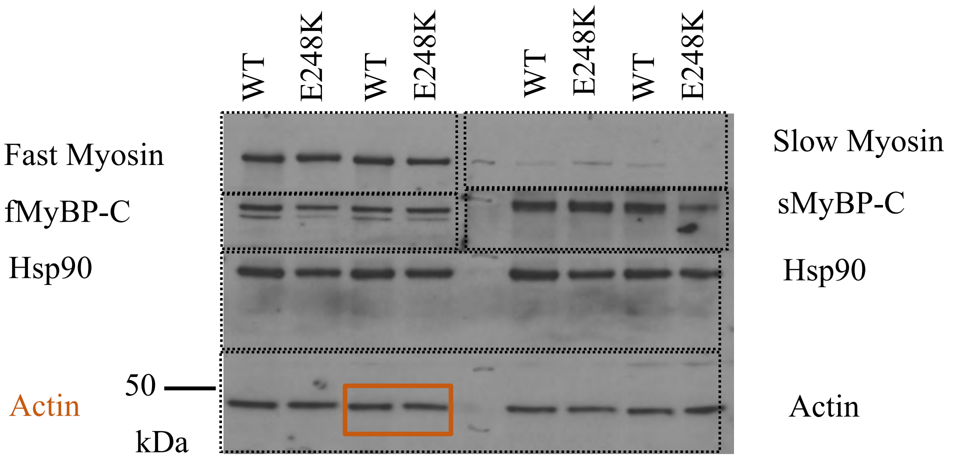

Original blots in Supplemental Fig 1B

2 weeks-Soleus

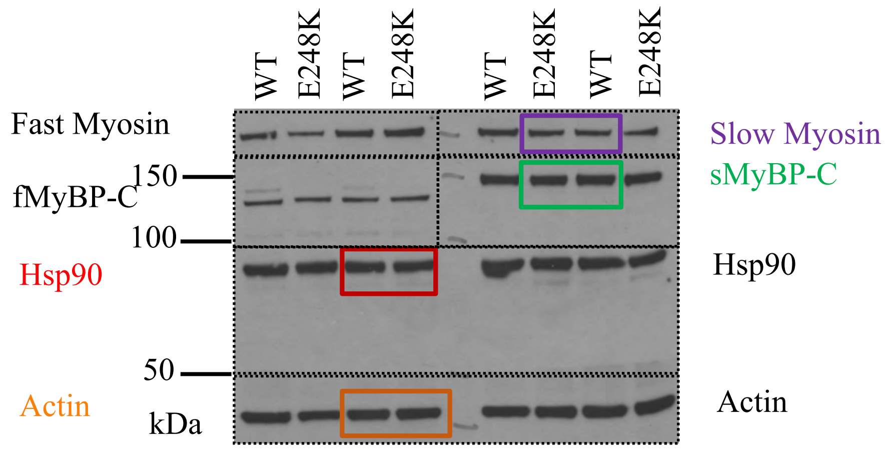

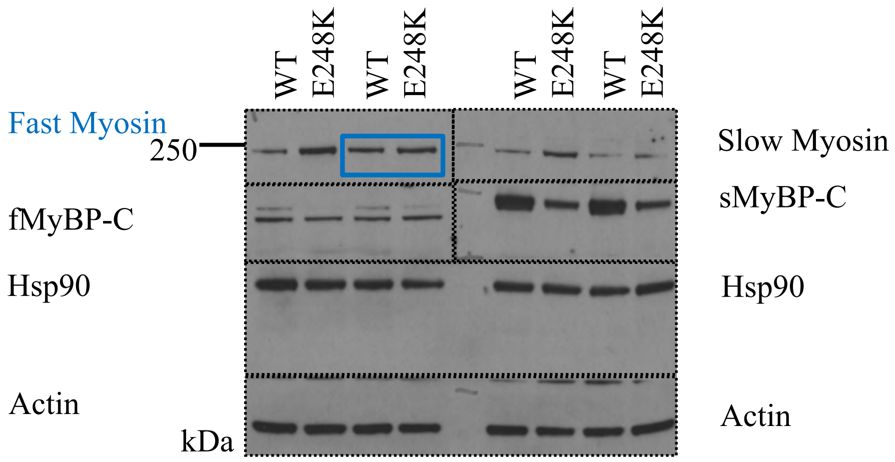

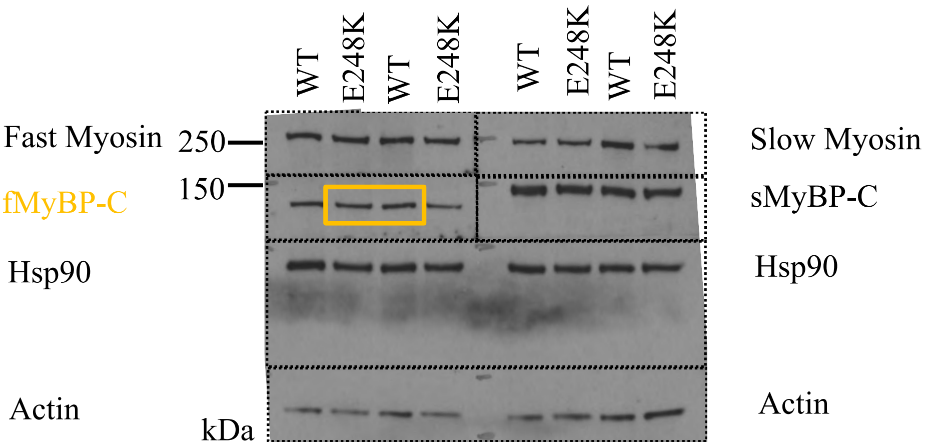

Original blots in Supplemental Fig 1C

2 weeks-Diaphragm

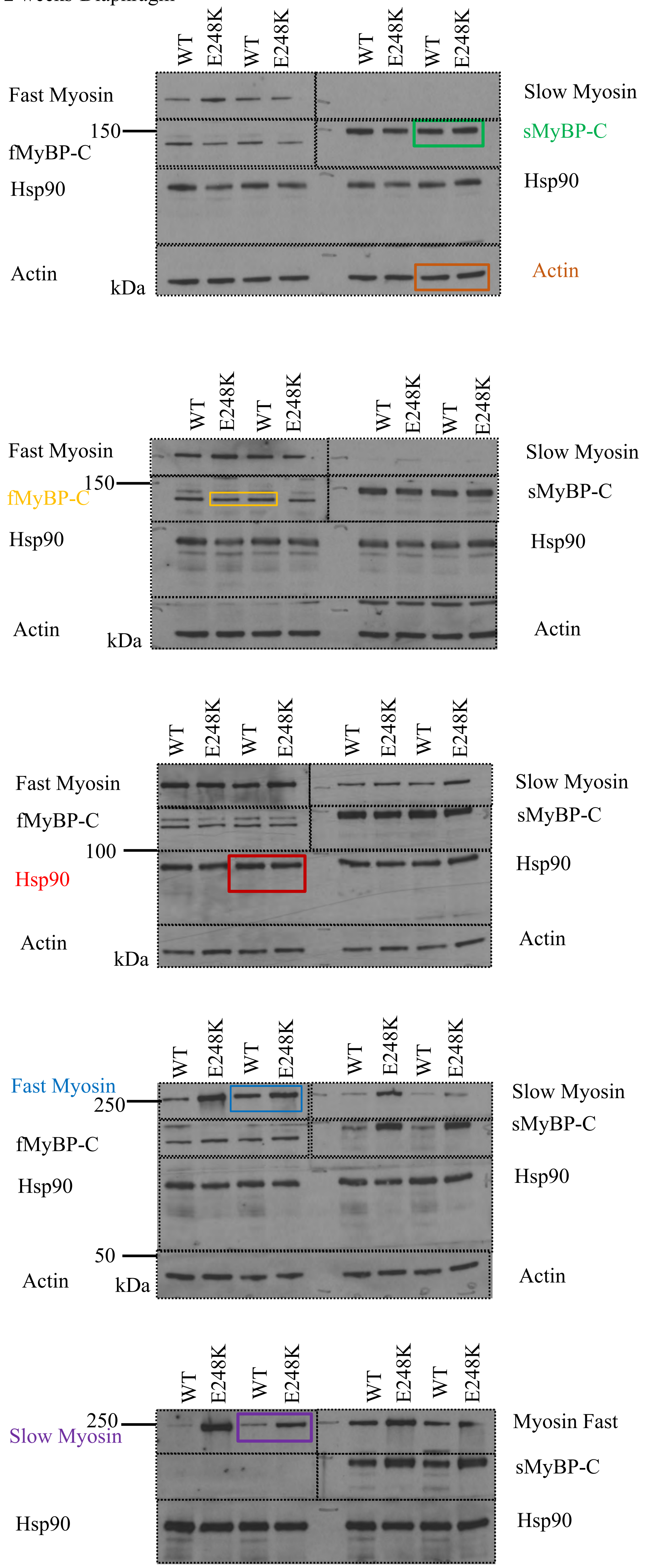

Original blots in Supplemental Fig 1D

4 week-EDL

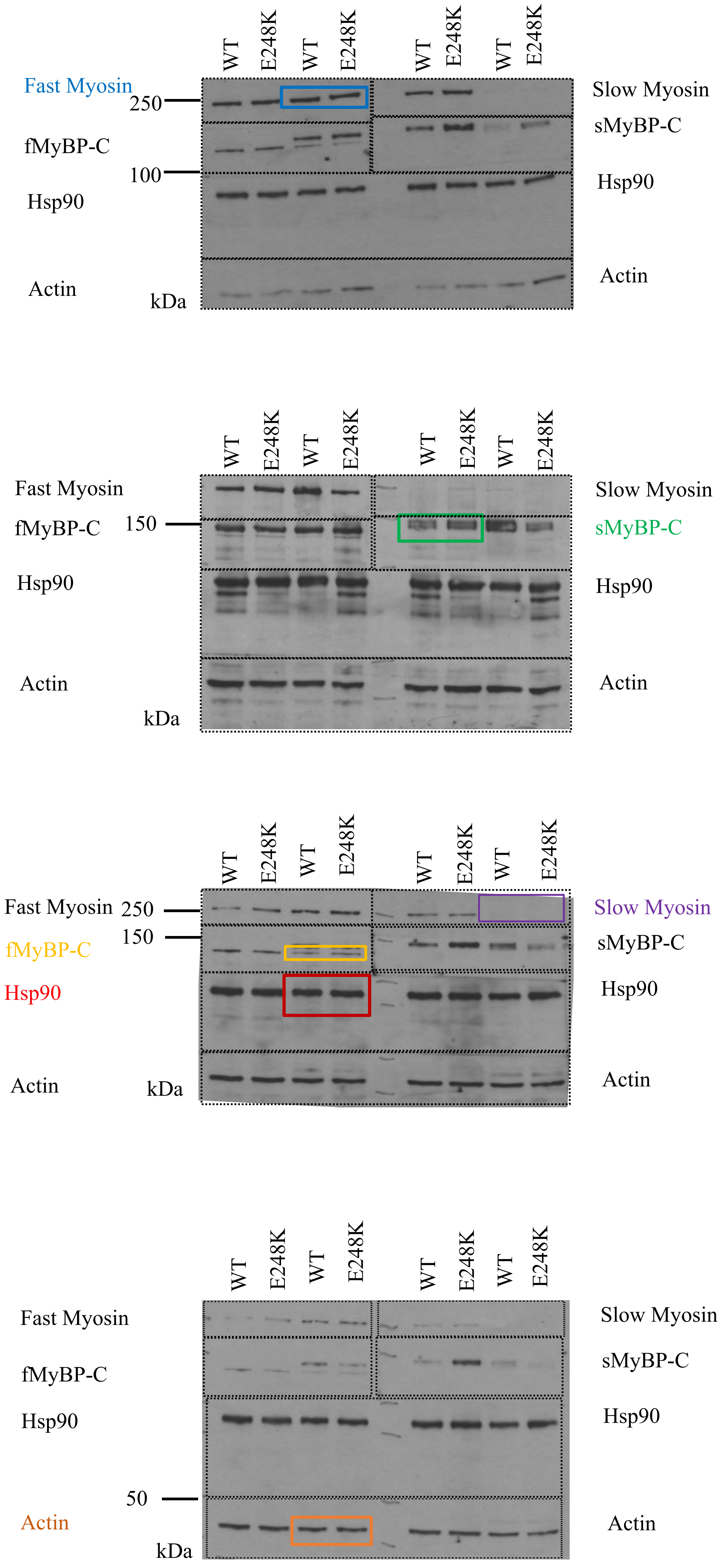

Original blots in Fig 3A

4 week-Soleus

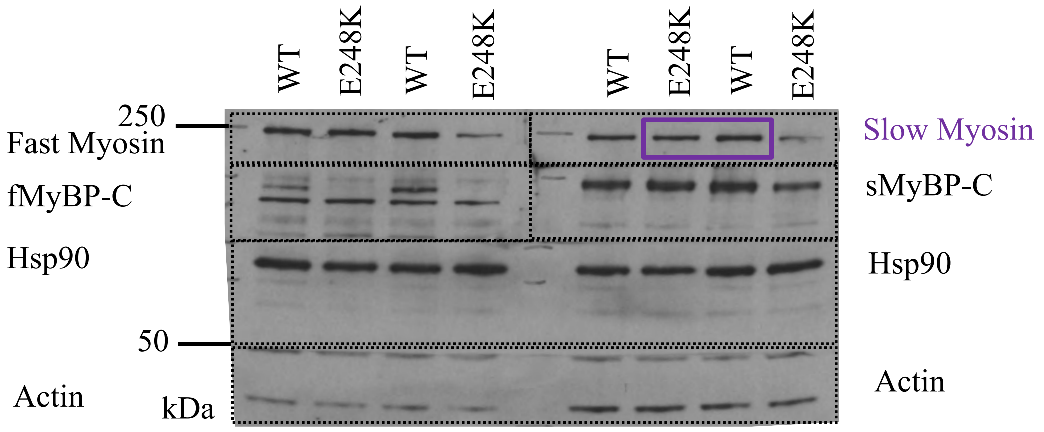

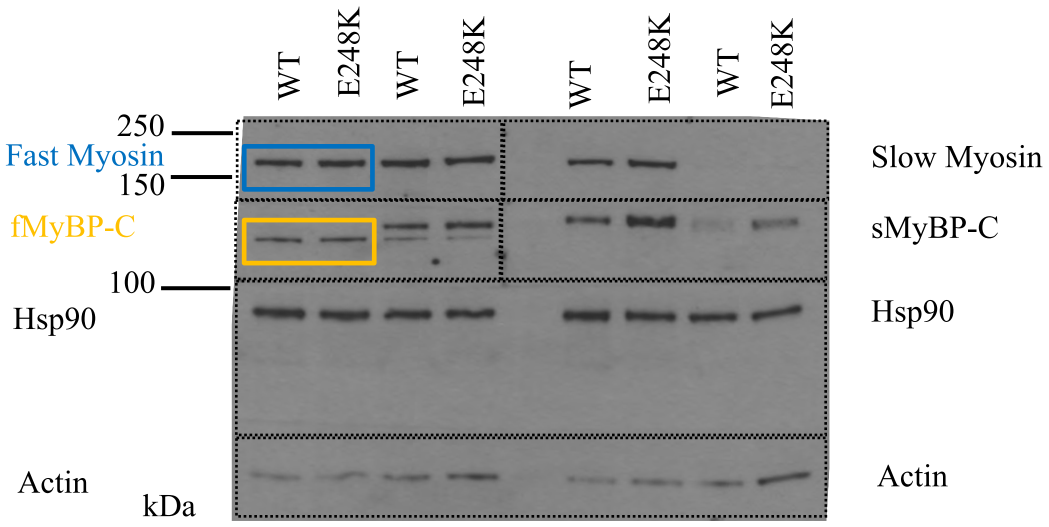

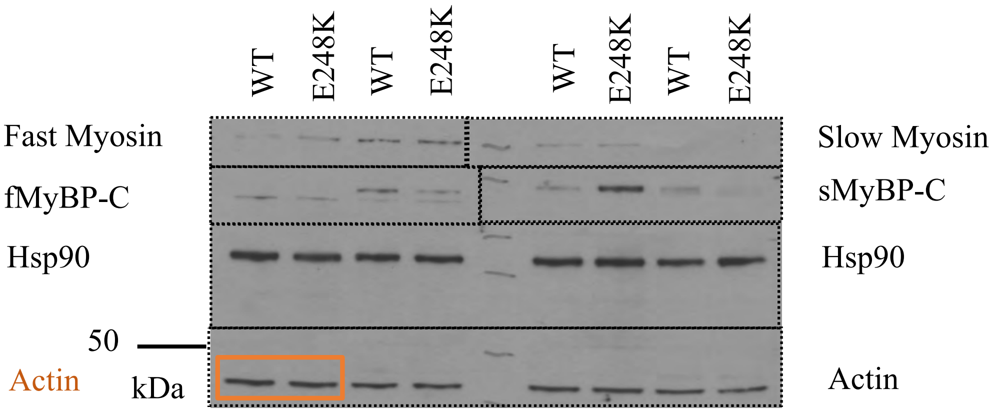

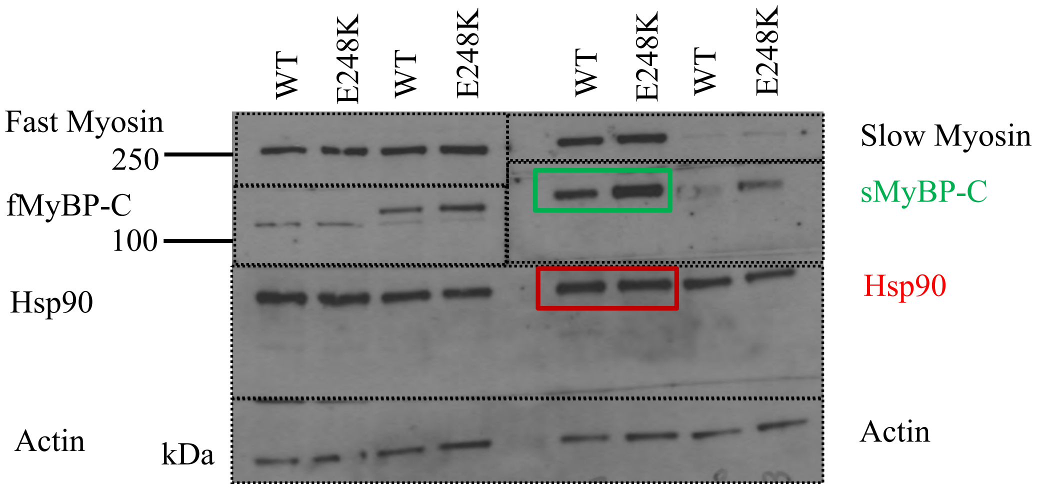

Original blots in Fig 3B

4 week-Diaphragm

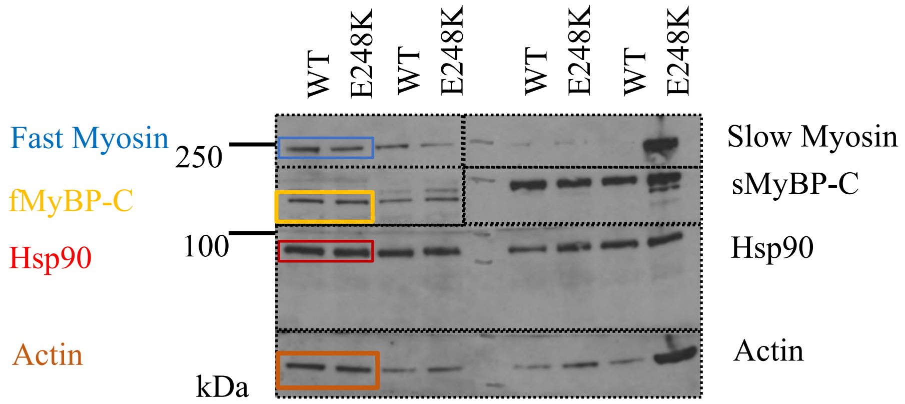

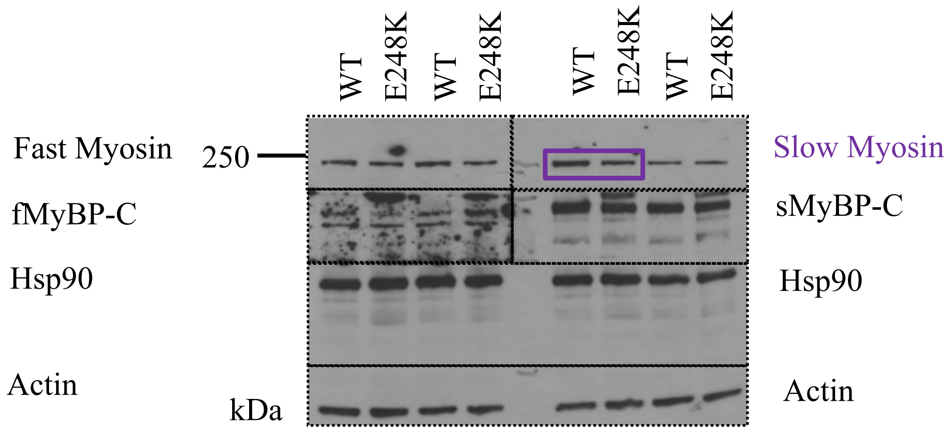

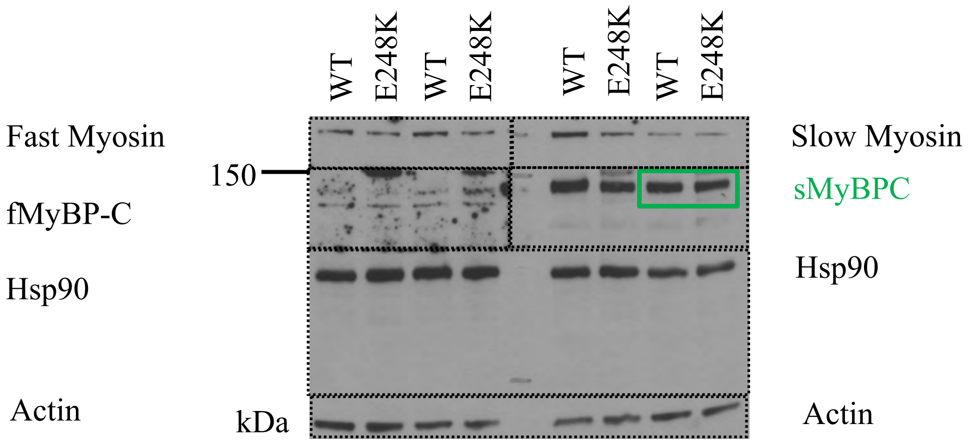

Original blots in Fig 3C
